# Examining the Flourishing Impacts of Repeated Visits to a Virtual Art Museum and the Role of Immersion

**DOI:** 10.3390/bs12120500

**Published:** 2022-12-07

**Authors:** Katherine N. Cotter, Damien L. Crone, Rebekah M. Rodriguez-Boerwinkle, Martin Boerwinkle, Paul J. Silvia, James O. Pawelski

**Affiliations:** 1 Positive Psychology Center, University of Pennsylvania, Philadelphia, PA 19104, USA; 2 Department of Psychology, Northeastern University, Boston, MA 02115, USA; 3 Department of Psychology, University of North Carolina at Greensboro, Greensboro, NC 27412, USA; 4 Department of Computer Science, University of North Carolina at Greensboro, Greensboro, NC 27412, USA

**Keywords:** virtual art, flourishing, immersion, well-being, art

## Abstract

Visiting art museums has been associated with a range of flourishing outcomes. However, there have been recent shifts towards increasing digital engagement with art, leading to a radical change in how people experience visual art. Given the now expansive virtual art viewing options, it is important to understand whether digital engagement can also lead to greater flourishing, and, if so, under what conditions. We examined the flourishing effects of viewing art in a virtual gallery in a pre-registered experiment comprising four sessions over four weeks, with varying viewing instructions designed to increase immersion. Participants were recruited from a USA representative sample on Prolific, resulting in a final sample of 687 participants. People were randomly assigned to one of nine experimental conditions. Eight art viewing conditions involved four 15 min virtual gallery visits with viewing instructions varying on two factors: slow-looking and immersive mindset framing. An active control condition involved reading about (but not viewing) art. Participants completed a battery of baseline flourishing measures in week 1, completed experimental art engagement sessions during weeks 1–4, and completed the battery again in week 5. While immersion levels were greater in the viewing conditions than the reading condition, growth in flourishing did not differ across condition. Exploratory analyses, however, showed that immersion during the gallery visits did predict some changes in specific facets of flourishing (e.g., engagement, meaning, autonomy satisfaction). We suggest a number of possible explanations for these null results and point to what is needed in future research.

## 1. Introduction

People across the world are currently in a crisis of health and well-being. Loneliness and social isolation are on the rise, experiences of depression, anxiety, and stress are increasing, and these effects are likely exacerbated by the COVID-19 pandemic [1,2]. As society becomes increasingly interested in non-medical means of addressing health and flourishing concerns (e.g., social prescribing [3,4]), it is unsurprising that research would turn to examining how our leisure time activities contribute to our flourishing [5].

Engaging with the arts (e.g., visual art, music, theatre, dance, etc.) is a common leisure activity that has increasingly been examined in recent years, with arts engagement shown to be a consistent presence in people’s lives around the world [6,7,8,9,10]. Recent research has found a range of flourishing benefits associated with arts engagement (see [11] for review). Greater or more frequent engagement with the arts has been related to lower risks of mortality [12], mental health conditions (e.g., depression, anxiety, or dementia [6,13,14,15]), reduced loneliness [16], and greater subjective health [6,17,18].

Unsurprisingly, consideration of the impacts of the arts is not new. There is a long history of philosophical considerations of aesthetic experiences and art engagement [19,20]. The arts have been seen as a way to feel moved or elevated [21], and experience enlightenment [22] or transformation [22,23]. Study of these impacts, judgements, and reactions has been guided by the field of empirical aesthetics [24] and numerous theoretical models have been introduced to guide the understanding of art engagement and aesthetic responses [25,26,27,28].

Among the different artistic disciplines, visual art (along with music) has been found to be one of the most common forms of people’s personally significant forms of engagement with the arts [29]. Within the museum studies field, scholars have investigated the impacts of visiting art museums on well-being outcomes, such as stress [30], emotional well-being [31], and feelings of connection [32,33]. Within psychology, meanwhile, scholars have examined the role cultural engagement plays in depression [14], anxiety [13], and global well-being [11]. Cotter and Pawelski [34] identified four domains of flourishing associated with art museum engagement: mental and physical health, subjective health and well-being, emotional well-being, and social connection. Specifically, visiting art museums has been shown to reduce stress [35,36], anxiety [37], and social disconnection [38], and to increase positive emotion [31,39,40], subjective well-being [36,41], and quality of life [18,42].

In addition to the consistent associations found between art engagement and flourishing outcomes, some programs explicitly focus on cultivating flourishing through art engagement. In the United Kingdom, health providers are able to write prescriptions for engaging with the arts to alleviate health conditions and to target social determinants of health (e.g., isolation [43]). This arts on prescription model has provided many benefits to participants, including reducing the need for general practitioner and hospital visits. Further, arts institutions themselves have begun to offer programs directly targeting flourishing in their visitors (e.g., *Meet Me at MoMA* [44]) in line with a recent survey finding that art museum professionals feel that art museums should be doing more to enhance the well-being of their visitors [45].

However, recently there has been a rapid increase in the ways in which people can engage with art online. Given the well-documented impacts on in-person art engagement and flourishing, it is likely there are also benefits to digital art engagement; however, few studies have addressed this question. Further, although the links between art engagement and flourishing are well-established, less is known about what qualities of the art engagement experience underly this relationship. The present research examined how online art engagement via visits to a virtual art gallery impacts flourishing and how a quality of the experience theorized to be related to flourishing [46,47,48]—immersion during art viewing—may influence flourishing benefits.

Recently, there has been an increase in the availability of digital options for viewing and engaging with art. In the wake of the COVID-19 pandemic, many art museums pivoted from predominantly in-person engagement with visitors to virtual engagement, developing new ways in which to engage their audiences [49,50,51]. Although few studies have examined the flourishing benefits of digital art engagement, these initial studies do suggest that digital art engagement also positively affects flourishing. Viewing art on a computer has been associated with lowering negative mood [52,53], increasing positive mood [52,54], reducing pain levels [55], and elevating overall well-being [53]. Given the well-documented impact of in-person art engagement on flourishing, we examine here whether digital art engagement has similar effects. We also examined the role of immersion (theorized to be related to flourishing, [46,47,48]). 

### 1.1. Cultivating Flourishing through Art

The recent RAISE model [46,47,48] has proposed five mechanisms to explain the relationship between art engagement and flourishing: (1) *Reflection*: intentionally developing, reinforcing, or discarding habits, values, or worldviews; (2) *Acquisition:* processes involved in gaining skills, experience, or knowledge through art engagement; (3) *Immersion*: when attention is captured by the art engagement, feeling flow, or feeling “carried away”; (4) *Socialization:* bonding or having conversations during art engagement; and (5) *Expression:* expressing oneself in creative ways. Of these mechanisms, immersion has been suggested to be the “gateway” mechanism that enables the others to occur [47].

Immersion has been linked to a range of flourishing benefits during engagement with differing artforms, including positive psychological and physiological states [52,56,57,58,59,60] and subjective well-being [61]. A common way in which immersion during arts engagement has been examined is through flow experiences (i.e., being fully immersed or absorbed in an activity and finding the activity rewarding), which can be considered the ideal form of immersion [47]. Flow has been associated with several flourishing outcomes, such as experiencing higher subjective well-being [58,62], fulfillment of psychological needs [62], and positive emotion [63].

Within the context of art viewing, one way in which we may observe immersion is through the practice of slow looking. In contrast to average viewing times of individual artworks in art museum contexts being less than one minute [64,65], slow looking involves spending an extended amount of time viewing a single artwork [66] and aligns with the conceptual definition of immersion in which a person’s attention is captured [47]. Further, slow looking is a practice commonly emphasized by art museum professionals, with over 1500 institutions engaging in the yearly Slow Art Day [67]. In one study, participants engaged in three 3 min online slow looking exercises in which they were presented with one of three types of information about the art—a control passage, a passage encouraging mindfulness, and a passage providing art historical information [54]. Following the slow looking in all three conditions, people felt more pleasant and relaxed; however, there was no non-slow-looking control. A subsequent study examined two forms of slow-looking (mindful vs. curious [52]) as well as a non-slow-looking control condition and found that degree to which people were immersed in this experience predicted greater intensity of positive and aesthetic emotions (e.g., awe, moved, chills) and lower intensity of negative emotions. It is important to note, however, that the three conditions did not differ in immersion levels, though this may have been due an insufficient number of artworks for the instructed viewing time and insufficient instructions for slow-looking.

Simply spending more time in front of an artwork is likely not the only important factor to examine as a predictor of immersion. Contextual factors of art viewing also influence the nature of the experience [26,27,28,68,69]. For example, being told about an artist’s intentions for the work [70] or the effort required to produce the artwork [71] influences people’s judgments about the artwork, including the degree to which they liked the work. Further contextual information may be more useful in certain situations, such as providing titles [72] or descriptive information [73] to better understand abstract but not representational artworks.

In addition to the influence of the context in which art is presented, the mental context of the viewer has to be examined. There are well-documented influences of several individual differences (e.g., art expertise [74,75]; openness to experience [76,77]) on art viewing experiences; however, less work has examined how momentary states or mental contexts shape art engagement. Art can evoke a range of emotions (see [28]), but mood prior to viewing an object has been shown to impact aesthetic responses to product designs [78,79] and photographs [80]. Other studies have induced mindful states through meditation prior to art viewing [54,81], yielding deeper aesthetic experiences compared to a control group [81]. This small collection of studies suggests that the mental context during art viewing can impact subsequent engagement with an artwork.

### 1.2. Present Research

Although, as mentioned, in-person art museum visits enhance flourishing [34], why this occurs is not clear. We examined the flourishing effects of a series of virtual art museum visits focusing on the possibly differential impacts of immersive art viewing practices. We tested the effects on flourishing of both slow looking instructions and three kinds of immersive viewing instructions, as detailed below. Both slow looking and the specific immersive mind set framings implemented in this study are aligned with the principles of immersion identified in the RAISE model [46,47,48].

For the immersive mindset framing factor, there were four sets of framing instructions—one for mindful looking (i.e., being aware of the experience of viewing the artwork), one for curious looking (i.e., generating questions about the artist and art), one for social looking (i.e., relating the artistic contents to personally significant relationships), and one that did not provide any specific viewing instructions that served as a control condition. The mindful looking condition reflects shifts within art museums to center visitor reactions and art-viewing experiences [82], the curious looking condition aligns with many art educational programs that emphasize details about the artwork or its artist [83,84], and the social looking condition reflects that in many cases people do not visit art museums by themselves [85] and may use these visits as an opportunity to refine or broaden their social identities [47]. This was a fully crossed experimental design with each immersive mindset framing occurring both with and without slow looking instructions. There non-gallery reading condition involving reading short art history narratives with no art images.

We assessed a range of flourishing outcomes: depression, anxiety, stress, overall thriving, and eighteen dimensions of well-being. We also included measures of autonomy, competence, and relatedness for use in exploratory analyses. For the flourishing measures, we had five sets of predictions:

1. Participants in eight virtual gallery conditions should show greater immersion than those in the non-gallery reading condition. Both slow looking and immersive mindset framing instructions should lead to greater immersion compared to no slow looking or immersive framing instructions.
2. Participants in the eight virtual gallery conditions should show greater gains in flourishing than the non-gallery reading condition.
3. Participants in the slow-looking condition should show greater gains in flourishing than those in the condition with no specific looking instructions.
4. Participants in the three conditions with immersive mindset framings should show greater gains in flourishing than those in conditions with no immersive mindset framing. We did not have predictions regarding differences between the three immersive mindset framing conditions.
5. Participants in conditions with both an immersive mindset framing and slow-looking instructions should show greater gains in flourishing than those in the condition with no specific looking instructions. We did not have predictions regarding differences between the three immersive mindset framing conditions.

## 2. Method

The data collection and analysis plan was pre-registered on the OSF (The full pre-registration can be viewed at https://osf.io/3erds/?view_only=048fbf3effef473c8bca905c11aa2bef).

### 2.1. Initial Screening

**Participants.** A sample of 2000 participants was recruited from Prolific. The sample was representative of United States adults with respect to gender, age, and race/ethnicity (see Table 1 for demographic information). The representative sample was obtained through Prolific’s representative sampling tools (See https://researcher-help.prolific.co/hc/en-gb/articles/360019236753-Representative-samples for additional details regarding Prolific’s representative sampling processes), which stratifies the intended sample size across age, gender, and race/ethnicity using census data from the US Census Bureau to approximate as closely as possible the representation of demographic groups invited to participate in the research. The purpose of recruiting this sample was to ensure appropriate technological compatibility of participant computers to enroll 1200 participants in the main study. Participants were paid $0.85 for the initial screening session.

**Procedures.** After providing informed consent and completing a brief demographic questionnaire, participants completed a short visit to a virtual art gallery (described below). Participants were told that they would have three minutes to spend within the virtual gallery. In order to activate the gallery, they clicked on a thumbnail in the survey to make the virtual gallery full screen. Participants navigated through the gallery using the arrow keys on the keyboard and using their mouse to change their viewpoint. Participants were also able to make individual artworks full screen and were instructed to make their favorite artwork full screen by clicking on it.

**Invitation to main study.** In order to qualify for the main study, participants needed to have been able to enter the virtual gallery in full screen, navigate through the gallery for a minimum of 90 s, and enter both rooms of the gallery (The pre-registered inclusion criteria also stated the participants were required to make their favorite artwork full screen. However, due to technical and instructional difficulties that were addressed prior to the main study experienced by many participants, we did not exclude participants from the main study who did not make an artwork full screen). A total of 1479 participants met these criteria.

**Gallery apparatus.** The virtual gallery was created using the Open Gallery for Arts Research (OGAR [86]). This tool allows for the creation of custom gallery floorplans and displays art images with which participants can interact. During data collection, participant behavior in the gallery (i.e., location in the gallery, what is being viewed, viewing individual works in full screen, time spent within the gallery) was tracked and recorded for later analysis.

For the initial screening session, a two-room gallery was created that displayed 10 artworks (see Supplementary Table S1). (Artworks included in the screening gallery were not used in the main study galleries). The artworks varied in style and content. From OGAR, we obtained time, in milliseconds, spent within the gallery, how long each artwork was viewed, which artwork was viewed for the longest time, and, for the artwork viewed the longest, how long that individual work was viewed. From these individual measurements, we also calculated how long people spent looking, on average, at individual artworks, and the total time spent viewing art (versus non-art targets, such as the wall, floor, or ceiling of the virtual gallery).

**Artwork selection.** All artworks used in the initial screening and main study were sampled from the Philadelphia Museum of Art (PMA) catalogue. We first scraped all 5488 paintings from the PMA online catalogue available at the time of study design, excluding all paintings that (1) the PMA asked us not to use in our research, (2) had an aspect ratio outside of the range 3/4 or 4/3, or (3) had a height and/or width of less than 500 pixels. This left a population of 1107 paintings from which to select our sample.

Our goal was to select a sample of artworks with maximally diverse visual content. To achieve this, we relied on computer vision-based description of the artworks, and an algorithmic sampling procedure that maximizes sample diversity based on this computer vision-based description. For the first step in this process, we used a neural network-based computer vision model (VGG16) to provide a quantitative description of the content of each painting. Then, to sample from the population of paintings, we (1) constructed an image-by-image distance matrix, representing the Euclidean distance of each image from one another based on the high-dimensional description provided by VGG16, (2) used a genetic algorithm to draw a sample of 180 paintings that is described by the dendrogram with the greatest cumulative branch height (i.e., the sum of the height of each branch), and (3) used the 180 paintings drawn through this procedure to populate the study galleries. Note that maximizing cumulative dendrogram branch height is functionally similar to the objective of maximizing the minimum inter-stimulus distance, while also being sensitive to higher-order clustering. The algorithmic artwork selection was performed using the R package gasample [87]. The 180 images were them manually screened to exclude any that were not images of an individual work (e.g., included a frame, contained multiple works, were not paintings or drawings), which reduced the usable number of images to 161.

### 2.2. Main Study

**Participants.** A total of 1479 participants (see Table 1) from the screening study were invited to participate in the main study, and 1200 participants enrolled. Participants were paid $5 for completing each of five sessions and an additional $5 bonus for completing all five sessions. To be retained for the final sample, participants needed to complete both the pre- and post-test flourishing measures, have low scores on inconsistent responding and directed responding questions checking for inattentive or careless responding, and complete at least three valid experimental sessions. For participants in the gallery conditions, a valid experimental session required walking at least 10 m within the gallery, moving the mouse to indicate changing their viewpoint, entering at least two gallery rooms, spending at least 10 min within the gallery, and not providing a nonsensical answer to an open-ended question describing their gallery experience. Participants in slow-looking conditions also needed to spend a minimum of 6 min viewing a single artwork. A valid experimental session for participants in the non-gallery control condition required answering at least two of three reading comprehension questions correctly.

The final sample (out of the 1200 who enrolled) consisted of 687 participants meeting all inclusion requirements (see Table 1). Participants were predominantly White (80.05%), middle-aged (*M* = 47.31, *SD* = 14.96), female (50.95%), and educated (60.55% with a Bachelors degree or greater).

**Procedures.** The study took place over the course of 5 weeks, with one research session per week. In week 1, participants completed baseline flourishing measures and individual differences surveys. Following the baseline measures, participants were randomly assigned to one of nine experimental conditions and completed their first experimental session. Following the experimental session, they completed additional measures about their experience. In weeks 2–4, participants completed additional experimental sessions and measures about each week’s experience. In the final session during week 5, participants completed post-test flourishing measures. 

**Measures.** Three flourishing measures were administered prior to any art engagement and one week following the final art engagement session. The *Comprehensive Inventory of Thriving* (CIT [88]) is a 54-item measure assessing well-being across 7 domains (i.e., relationships, engagement, mastery, autonomy, meaning, optimism, and subjective well-being) and contains 18 specific subscales within these domains. Items were rated on a 5 pt. Likert scale (*Strongly Disagree* to *Strongly Agree*). The *Depression Anxiety Stress Scales* (DASS-21 [89]) is a 21-item measure assessing symptoms of depression and anxiety and feelings of stress. Items were rated on a 4 pt. Likert scale (*Did not apply to me at all* to *Applied to me very much or most of the time*). The *Balanced Measure of Psychological Needs* [90] is an 18-item measure assessing satisfaction and dissatisfaction with self-determination theory domains (i.e., autonomy, competence, and relatedness). Items were rated on a 5 pt. Likert scale (*Strongly Disagree* to *Strongly Agree*). Additional individual measures, including measures of personality, art interest, and people’s broader engagement with the arts and humanities were also collected during people’s first session. These measures are unrelated to the manuscript’s present aim, however, and are not discussed further.

Following each of the art engagement sessions, participants completed a four-item measure of immersion assessing the degree to which they lost track of time, got lost in thought, were focused on viewing the art/reading about art, and felt the experience was rewarding. Items were rated on a 7 pt. Likert scale (*Strongly Disagree* to *Strongly Agree*). Participants in the gallery conditions also responded to an open-ended prompt about their experiences within the virtual gallery.

**Figure 1:**
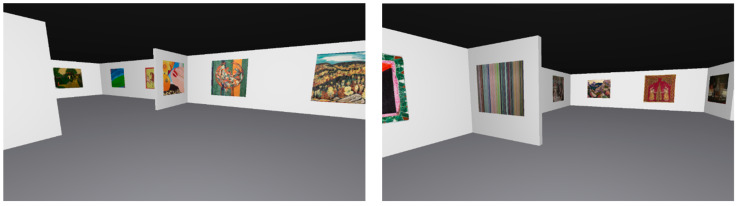
Screenshots from OGAR galleries created for the present research.

**Gallery construction.** The galleries were constructed in OGAR with an identical four-room layout (see Figure 1 for examples). Each gallery contained 30 artworks drawn from the pool of 161 artworks described above. Specific artworks were used in only one gallery, and artworks were selected to balance the number of representational and abstract works within each gallery and to vary the contents of artworks within each gallery (see Supplementary Table S1 for a list of artworks used in each gallery). For each gallery, we obtained time (in msec) spent in the gallery, time spent viewing each artwork, and the name of the artwork viewed the longest. From these individual measurements, we also calculated how long people spent looking, on average, at individual artworks, and total time spent viewing art (versus non-art targets, such as the wall, floor, or ceiling of the virtual gallery). A different gallery was used in each week’s viewing session so as to present new works each week, and all experimental conditions were exposed to the same artworks each week.

**Experimental conditions.** Participants were randomly assigned to one of nine conditions for the duration of the study (see Table 2). Eight conditions involved a virtual art gallery session with viewing instructions varying on two factors: slow-looking and framing. The slow-looking factor had two variants—a no slow-looking variant in which participants were free to view individual artworks for as long as they wished (four conditions, which vary in framing), and a slow-looking variant in which participants were instructed to select a single artwork to view for 10 min (four conditions, which vary in framing). The framing factor had four variants—a control framing in which participants were told to navigate the gallery and view art how they would like to (2 conditions, which varied in slow looking instructions), a mindful framing in which participants were told to reflect on their reactions to the work and to not judge these reactions (2 conditions, which varied in slow looking instructions), a curious framing in which participants were told to ask questions about the artwork, the artist, and the intended interpretation (2 conditions, which varied in slow looking instructions), and a social framing in which participants were asked to think about how the artwork connected to significant relationships in their lives (2 conditions, which varied in slow looking instructions; see Table 2 for exact instruction language). In three of the experimental conditions, participants were given both slow looking and immersive mindset framing instructions. The final experimental condition was a non-gallery control condition in which participants read about topics in art history (*n* = 100; see https://osf.io/r7pgy/ for exact passages) and answered three multiple choice questions to test their understanding.

## 3. Results

Descriptive statistics are available in Supplementary Tables S2 and S3. Overall, the participants showed very little change in flourishing measures between the pre-test and post-test measurements (see Supplementary Figure S1).

### 3.1. Did the Experimental Conditions Differ in Immersion?

We first examined whether the conditions differed in their levels of immersion via multilevel models using the lmerTest and performance package in R (version 4.20; [91,92,93]; see Table 3). The intraclass correlation coefficient for immersion was 0.67, indicating that 67% of the variability in people’s immersion responses can be attributed to person-level factors. People who were assigned to a gallery condition experienced greater immersion than those assigned to the non-gallery reading control (*b* = 0.66, *p* < 0.001). However, immersion levels among the gallery conditions did not differ from one another. Thus, our instructional manipulations were not successful in increasing levels of immersion.

### 3.2. Does Viewing Art Improve Flourishing More Than Reading about Art?

Using multiple regression, we next examined whether the gallery conditions and the non-gallery control differed in flourishing using the post-test measurements (i.e., Comprehensive Inventory of Thriving, Depression Anxiety Stress Scales, and Balanced Measure of Psychological Needs) as the outcome variables and pre-test measurements and an indicator variable for condition (Gallery: 1, Non-Gallery: 0) as predictors. Pre-test measurements were z-score standardized and post-test measurements were standardized to the pre-test scale—the transformed post-test scores thus represented change in pre-test standard deviation units. These analyses were run in R using the ggplot2, lmtest, and sensemakr packages [94,95,96] (see Figure 1 and Supplementary Table S4).

**Figure 2:**
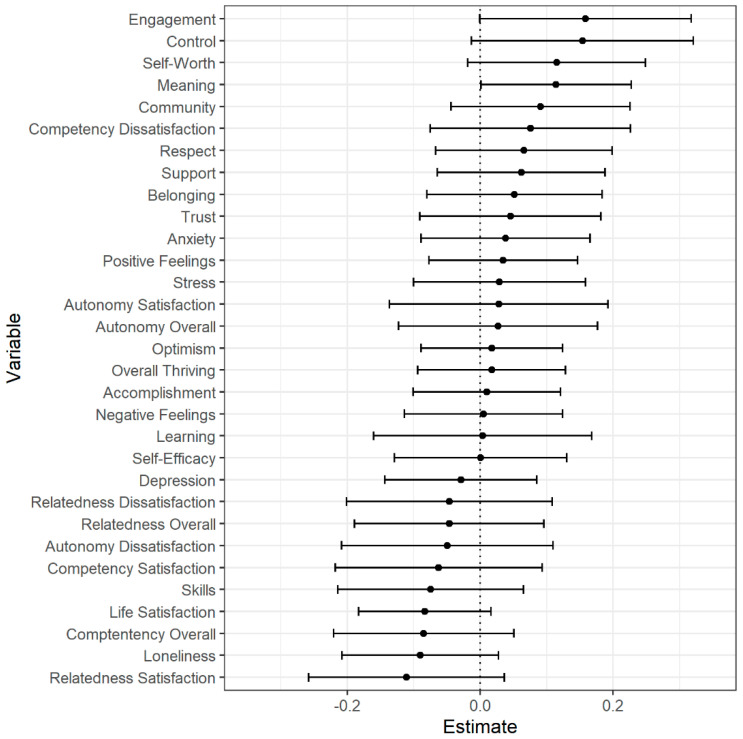
Differences in post-intervention well-being between Gallery (1) and Non-Gallery Reading (0) conditions. Note. Estimates represent regression weights (controlling for pre-intervention well-being) in pre-intervention standard deviation units. Values to the right of zero indicated higher well-being in the Gallery conditions. Error bars represent 95% confidence intervals.

Unsurprisingly, pre-test flourishing was a strong predictor of post-test flourishing. The only factor on which the gallery conditions differed from the non-gallery reading condition was in meaning (*b* = 0.12, *p* = 0.04; see Figure 2), as measured by items assessing feeling one’s life has purpose and knowing what gives life meaning. Those in the gallery conditions showing a greater increase in meaning than those in the non-gallery reading control; however, this was a small effect (*f*^2^ = 0.01). Additional exploratory analyses examining the interaction between condition and pre-test flourishing can be found in Supplementary Table S5 and Supplementary Figure S2.

### 3.3. Does Slow Looking Improve Flourishing More Than Not Slow Looking?

**Figure 3:**
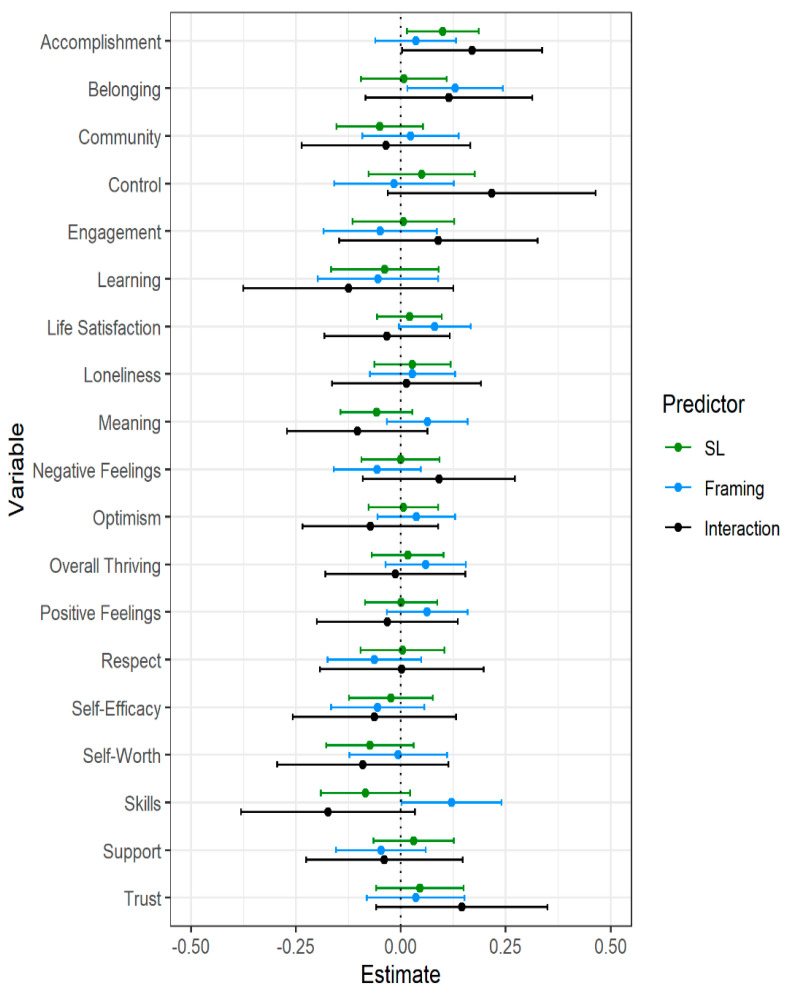

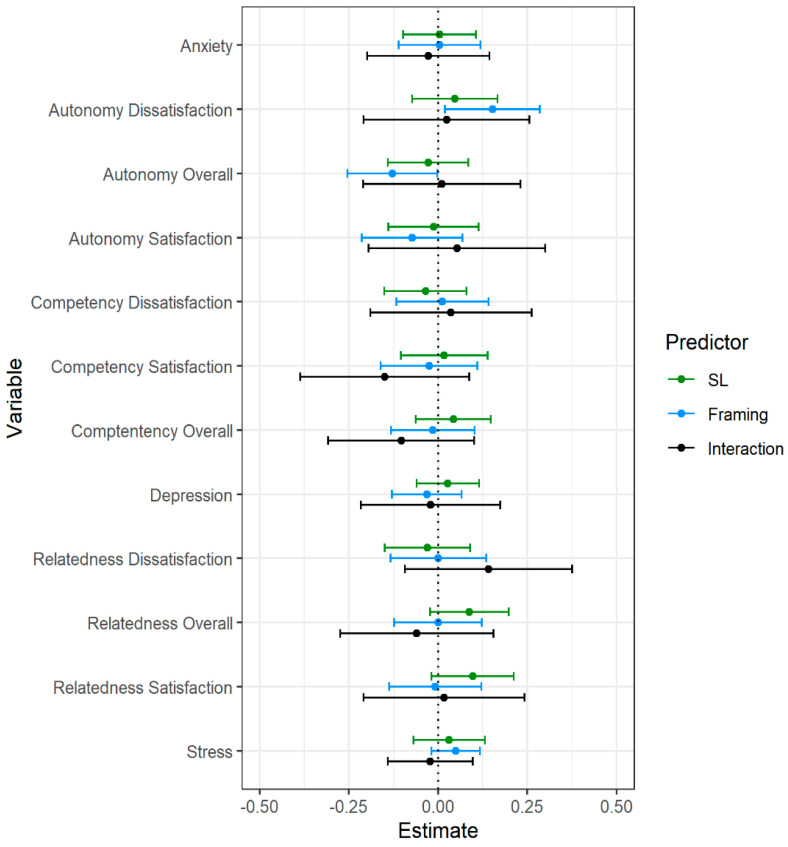
Differences in post-intervention well-being based on Slow-Looking, Immersive Mindset Framing, and Slow-Looking × Framing interaction conditions. Note. Estimates represent regression weights (controlling for pre-intervention well-being) in pre-intervention standard deviation units. Error bars represent 95% confidence intervals.

Next, we used multiple regression to examine whether people who were instructed to engage in slow-looking (with or without immersive mindset framing instructions) differed in changes in flourishing compared to those who did not receive slow-looking instructions. These analyses did not include the reading control condition. Post-test measurements were used as the outcome variables with pre-test scores and an indicator variable for condition (Slow-Looking: 1, No Slow-Looking: 0) as predictors (see Figure 3 and Supplementary Table S6). The only factor on which these the slow looking instruction conditions differed was in accomplishment (*b* = 0.10, *p* = 0.02), as measured by items assessing feeling on track to achieving one’s dreams or fulfilling one’s life ambitions. Those in the slow looking conditions showing a greater increase in accomplishment than those not in the slow looking conditions; however, this was a small effect (*f*^2^ = 0.01). Additional exploratory analyses examining the interaction between condition and pre-test flourishing can be found in Supplementary Table S7 and Supplementary Figure S2.

### 3.4. Does an Immersive Mindset Framing Improve Flourishing More Than No Immersive Mindset Framing?

Next, we examined whether people who were provided immersive mindset framings (with or without immersive mindset framing instructions) differed in changes in flourishing compared to those who did not receive these framings. These analyses did not include the reading control condition. Post-test measurements were used as the outcome variables in the regression models with pre-test scores and an indicator variable for condition (Immersive Mindset Framing: 1, No Immersive Mindset Framing: 0) as predictors (see Figure 3 and Supplementary Table S8). There were no differences in flourishing between conditions with immersive mindset framing instructions and the condition without these instructions. There were a few differences between the participants presented with immersive mindset framings and those without these framing instructions. People presented with the immersive mindset framings showed greater increases in belonging (*b* = 0.12, *p* = 0.04, *f*^2^ = 0.01) and in autonomy dissatisfaction (*b* = 0.16, *p* = 0.02, *f*^2^ = 0.01), and greater decreases in overall autonomy (*b* = −0.14, *p* = 0.04, *f*^2^ = 0.01) than those not given these framings. However, all three relationships were small in magnitude. Additional exploratory analyses examining the interaction between condition and pre-test flourishing can be found in Supplementary Table S9 and Supplementary Figure S2.

### 3.5. Is There an Interaction between Slow Looking and Immersive Mindset Framing on Flourishing on Outcomes?

We next examined the interaction between slow-looking instructions and immersive mindset framings on flourishing. Post-test measurements were used as the outcome variables with pre-test scores and indicators variable for condition (Slow-Looking: 1, No Slow-Looking: 0; Immersive Mindset Framing: 1, No Immersive Mindset Framing: 0) and their interaction as predictors (see Figure 3 and Supplementary Table S10). Although pre-test flourishing scores continued to predict post-test flourishing, there were no significant interactions between the slow looking and immersive mindset framing factors. Additional exploratory analyses examining the interaction effects with pre-test flourishing can be found in Supplementary Table S11 and Supplementary Figure S2.

### 3.6. Exploratory Analyses

**Immersion.** Because neither the slow looking nor immersive mindset instructions, nor their combination, resulted in differential levels of immersion compared to the condition with no specific viewing instructions, we conducted a series of exploratory analyses on participants in the eight gallery conditions to better understand the relations between virtual gallery experiences and flourishing. These exploratory analyses focused on participants in the eight virtual gallery conditions.

**Figure 4:**
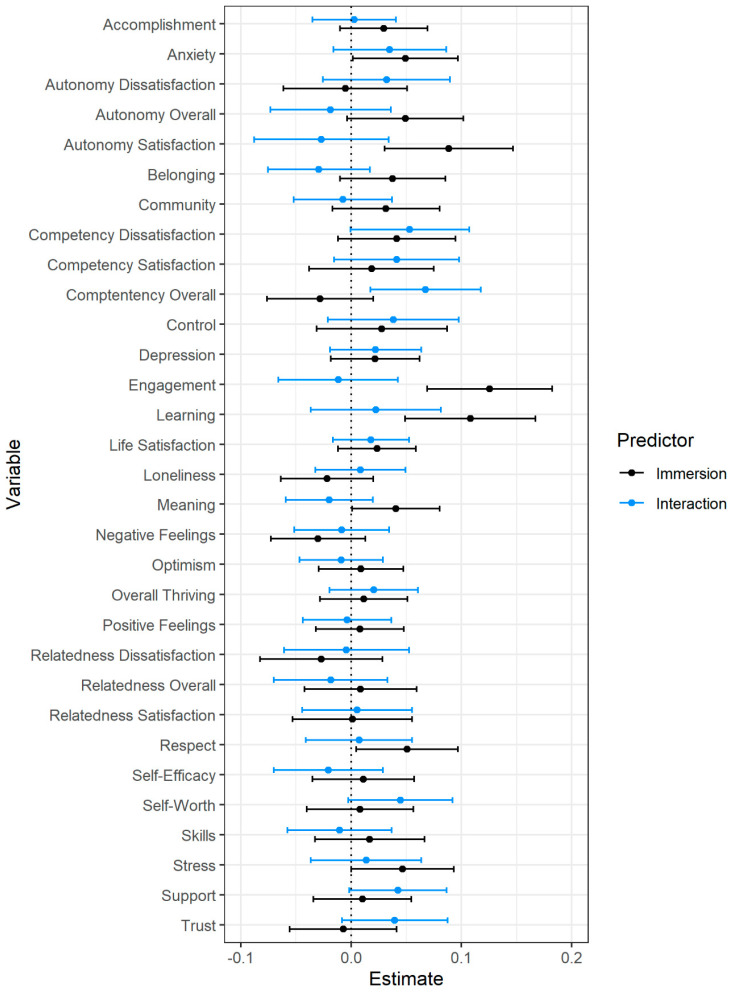
Latent immersion factor and interaction predicting post-intervention well-being. Note. Estimates represent regression weights (controlling for pre-intervention well-being) in pre-intervention standard deviation units. Error bars represent 95% confidence intervals.

Immersion scores from each of the four gallery visits were used as indicator variables in a confirmatory factor analysis to obtain an overall immersion value for each participant. These analyses were completed using the lavaan, lmtest, and sensemakr packages [94,96,97]. The confirmatory factor analysis fit the data well (χ^2^ = 1165.68, df = 6, *p* < 0.001, RMSEA = 0.07, 95% CI: [0.03, 0.13], SRMR = 0.01, CFI = 0.99, TLI = 0.98). We used the latent immersion values to predict post-test flourishing scores in addition to pre-test flourishing scores and the interaction between pre-test scores and immersion (see Figure 4 and Supplementary Table S12).

Like the pre-registered analyses, pre-test flourishing scores were the most consistent and strongest predictor of post-test flourishing. There were, however, several aspects of flourishing that were predicted by immersion during gallery visits. Higher levels of immersion were associated with greater increases in engagement (*b* = 0.13, *p* < 0.001, *f*^2^ = 0.03), learning (*b* = 0.11, *p* = 0.001, *f*^2^ = 0.02), meaning (*b* = 0.04, *p* = 0.05, *f*^2^ = 0.01), respect (*b* = 0.05, *p* = 0.03, *f*^2^ = 0.01), anxiety (*b* = 0.05, *p* = 0.03, *f*^2^ = 0.01), stress (*b* = 0.05, *p* = 0.05, *f*^2^ = 0.01), and autonomy satisfaction (*b* = 0.09, *p* = 0.003, *f*^2^ = 0.02); however, these associations were small in magnitude. One potential reason for the counterintuitive relation between increases in anxiety and stress and increases in immersion may be that immersive engagement is cognitively demanding, which may result in heightened anxiety and stress. Additionally, there was a significant interaction for overall competency—people low in competency at pre-test showed greater gains when they were less immersed during their gallery visits (see Figure 7, panel F).

**Qualitative analysis.** In our second set of exploratory analyses, we analyzed the open-ended descriptions of participants’ experiences provided at the end of each of their visits to the virtual gallery.

To summarize the open response data from each participant’s gallery visit, we employed topic modeling [98]. Topic modeling provides a relatively interpretable, bottom-up summary of a corpus in a functionally similar way to the use of exploratory factor analysis in the analysis of survey data. Formally, topic models are a form of latent variable model estimated from document-level word co-occurrences that describes a corpus of texts as a mixture of a discrete set of latent topics (e.g., Document 1 belongs mostly to Topic A, but also has a little bit of Topic B, and so-on). Topics, in turn are described as a probability distribution over the words in the corpus vocabulary, such that words that frequently co-occur with one another (at the document level) will be grouped together in the same topic (e.g., in the latent Topic A, the words “couch” and “sofa” might occur with a high probability, while words such as “bankruptcy” and “lobster” might occur with a low probability).

Text preprocessing was performed in part using the quanteda package [99]. Preprocessing proceeded as follows (see analysis code for further implementation details). We pooled all of participants’ responses across the multiple sessions into a single corpus. Texts were converted to lowercase, and punctuation was removed. We then removed responses with fewer than five words and removed words from the vocabulary if occurring in less than five texts Words below this threshold were first stemmed so that low-frequency terms (e.g., “activity” and “activities”) could be retained as a single stemmed term (e.g., “activ*”) if the word stem occurred with sufficient frequency. A custom set of 117 stopwords were also removed. After pruning the vocabulary, we then identified and joined high frequency multi-word phrases (e.g., “still life”). This produced a final corpus of 3050 text responses consisting of a vocabulary of 5957 words and phrases.

With this corpus, we then estimated a series of correlated topic models (using the stm package [100]) with varying numbers of topics (ranging from 10 to 30). After manually inspecting model quality across these different models (e.g., topic coherence and redundancy), we settled on a final model consisting of 14 topics used for the following analyses. Note that the topic model construction process was performed entirely independently of, and prior to, the inferential analyses reported below.

After the final set of topics were identified, these topics were then grouped into three categories: topics focused on participant descriptions of the art present during their visit (8 topics), topics focused on participants’ emotional states and feelings during the visit (4 topics), and topics related to navigation or qualities of the virtual gallery (2 topics). Based on these groupings, we were able to calculate the proportion of each response that belonged to these three categories. Across all responses, 56.52% (*SD* = 23.72%) of responses were art focused, 29.59% (*SD* = 20.57%) were feeling focused, and 13.89% (16.50%) were gallery or navigation focused. For the purposes of these analyses, we only focus on the proportions belonging to the art and feelings topic groupings.

**Figure 5:**
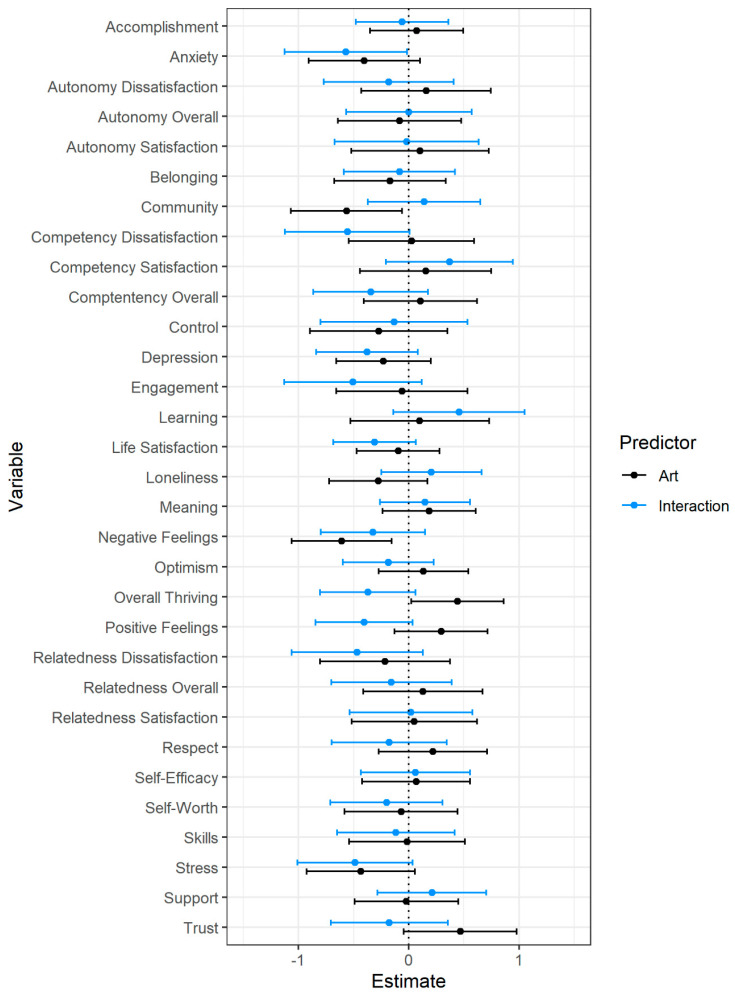
Latent art topic factor and interaction predicting post-intervention well-being. Note. Estimates represent regression weights (controlling for pre-intervention well-being) in pre-intervention standard deviation units. Error bars represent 95% confidence intervals.

**Figure 6:**
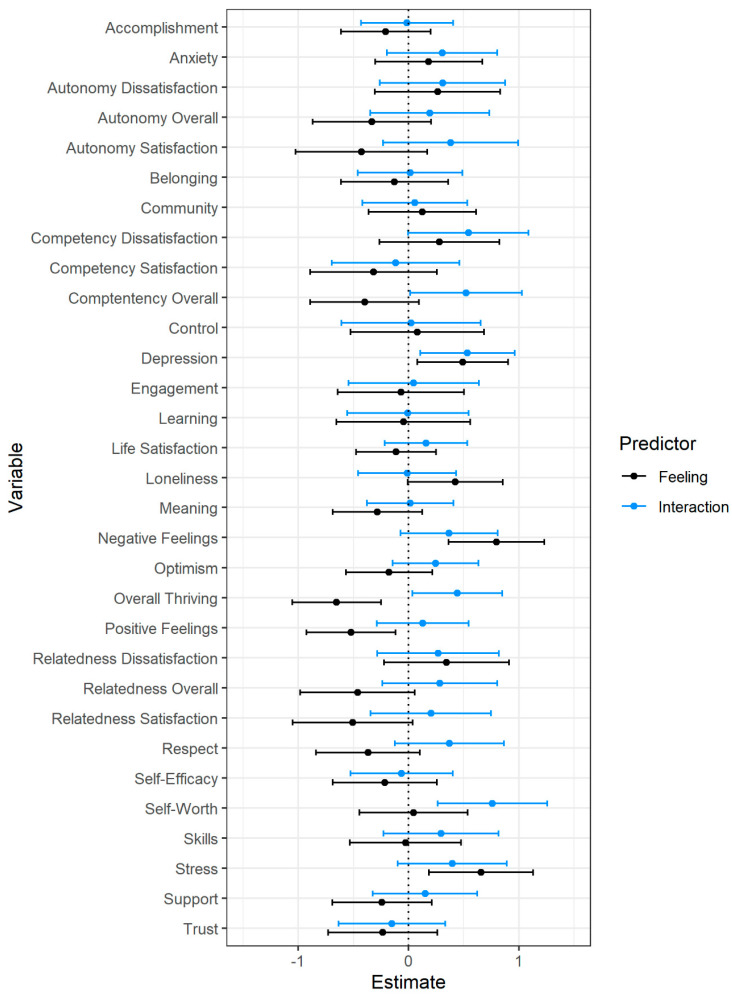
Latent feeling topic factor and interaction predicting post-intervention well-being. Note. Estimates represent regression weights (controlling for pre-intervention well-being) in pre-intervention standard deviation units. Error bars represent 95% confidence intervals.

Due to the dependency inherent in these topic groupings, analyses for the art and feelings topics were run separately. To obtain overall topic scores, separate confirmatory factor analyses using topic proportions for the four gallery visits as indicators were run to obtain overall latent topic scores for each participant. For each of the weekly gallery visits, the total proportion of each participant’s open-ended response that corresponded to art topics and feeling topics were used as the indicators for the factor analysis. The models fit the data well (Art: χ^2^ = 274.54, *df* = 6, *p* = < 0.001, RMSEA = 0.00, 95% CI: [0.00, 0.08], SRMR = 0.01, CFI = 0.99, TLI = 0.99; Feeling: χ^2^ = 261.25, *df* = 6, *p* = < 0.001, RMSEA = 0.00, 95% CI: [0.03, 0.06], SRMR = 0.01, CFI = 0.99, TLI = 0.99), and like the prior analyses, the post-test flourishing scores were predicted by the pre-test flourishing scores and the topic scores and the interaction between topic scores and pre-test flourishing were used as predictors (see Figure 5 and Figure 6 and Supplementary Tables S13 and S14).

**Figure 7:**
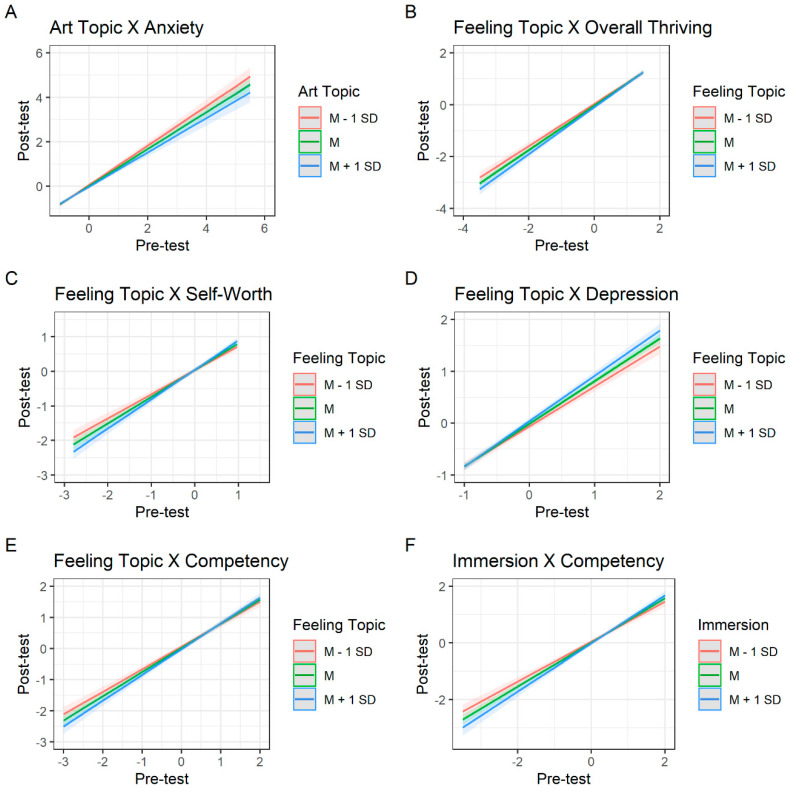
Interactions between pre-test flourishing, immersion, art topics, and feeling topics predicting post-test flourishing.

Although the two topic categories did not consistently predict flourishing outcomes, each were associated with two flourishing qualities. People whose open-ended responses featured greater descriptions of the artworks experienced greater decreases in negative emotions (*b* = −0.61, *p* = 0.01, *f*^2^ = 0.01) and sense of community (*b* = −0.56, *p* = 0.03, *f*^2^ = 0.01) and increases in overall thriving (*b* = 0.44, *p* = 0.04, *f*^2^ = 0.01). Additionally, the interaction between pre-test flourishing and art topic descriptions was significant for anxiety—people who were experiencing greater anxiety at pre-test showed greater reductions at post-test when their experience descriptions featured higher proportions of art-related topical content (see Figure 7, panel A).

Conversely, people whose open-ended responses featured greater descriptions of their emotional state and feelings experienced greater increases in negative emotion (*b* = 0.80, *p* = 0.001, *f*^2^ = 0.02), depression (*b* = 0.49, *p* = 0.02, *f*^2^ = 0.01), and stress (*b* = 0.65, *p* = 0.006, *f*^2^ = 0.01) and decreases in overall thriving (*b* = −0.65, *p* = 0.002, *f*^2^ = 0.02) and positive feelings (*b* = −0.52, *p* = 0.01, *f*^2^ = 0.01). Like all prior analyses, all effects were small in magnitude. Additionally, four interactions between feeling topics and pre-test flourishing were significant. People who were lower in overall thriving, competency, and self-worth at pre-test showed greater gains at post-test when their experience descriptions featured lower proportions of feeling topics (see Figure 7, panels B, C, and E). People who were higher in depression at pre-test showed greater reduction at post-test when their experience descriptions featured lower levels of feeling-related topical content (see Figure 7, panel D).

## 4. Discussion

The present research examined the impact on flourishing of four brief repeated visits to a virtual art gallery. We varied viewing instructions to examine the effects of slow looking and various kinds of immersive mindset framing compared to no viewing instructions. In a non-gallery control condition, participants did not view art but instead read about art. The gallery conditions were associated with greater immersion levels than the non-gallery condition. Contrary to hypothesis, however, neither the slow looking nor immersive mindset instructions, alone or in combination, were associated with greater flourishing compared to the condition with no viewing instruction. Instead, what did predict flourishing outcomes was pre-test flourishing scores. Exploratory analyses did demonstrate several effects (albeit small in magnitude) that are consistent with the links between immersion and flourishing outlined in recent theory (e.g., increases in engagement, meaning, and autonomy satisfaction [46,47]).

This study had a number of strengths. First, this study represents one of the first systematic examinations of impacts of engaging with art in a virtual gallery environment. Given the recent proliferation of curated online art engagement opportunities [49,50,51], understanding the psychological and flourishing impacts of these activities is a much needed avenue for continued research. Second, our sample was drawn from a representative (with respect to age, gender, and race/ethnicity) United States adult sample, a departure from many arts-based studies that often rely on student samples or on samples of individuals with prior interest in art engagement (e.g., art museum visitors), enhancing the generalizability of the present research. Finally, this is one of the first studies to examine the impacts of repeated art viewing sessions. Although population level studies suggest that repeated art engagement is important for flourishing benefits [6,13,14,15], there has been little experimental work examining the impacts of multiple viewing sessions as was done in the present research.

No study is without its limitations, however. We suggest several possible reasons and limitations for the lack of effects on flourishing of our interventions:

***Kinds of flourishing measures.*** One potential reason for the lack of links between immersion and flourishing is that people’s pre-test and post-test flourishing scores were remarkably stable. That is, there was no substantial change in flourishing for the immersion-related factors to predict that was not already accounted for by people’s pre-existing individual differences in flourishing. We asked for general, global assessments of each aspect of flourishing, and these may be less sensitive to change than more state-focused or context-specific measures such as emotion. For example, focusing on changes in emotional experiences, as has been previously documented as being influenced by art engagement, is a logical target, particularly as experiencing positive emotion during an activity is linked with continuing with that activity [101,102,103]. Because recurrent art engagement has been associated with a variety of flourishing benefits [14,15], impacting positive emotion in ways that encourages continued engagement with art may, over time, have flourishing benefits. Additionally, for flourishing outcomes that have study-specific connections (e.g., the theoretical connections between immersion and engagement), one approach may be to adapt existing measures to reflect the current moment (e.g., “During this visit, I felt energized”; “I was fully absorbed during the visit”) rather than more global statements (e.g., “In most activities I do, I feel energized”; “I get fully absorbed in activities I do”).

***Timing of flourishing measures.*** Our post-test flourishing measures were not administered immediately after art engagement but instead one week after the last viewing session. In-person museum studies have found a range of impacts on emotions immediately following a visit [104,105,106], with the most consistent findings showing increases in positive emotions [31,39,40,107]. Cotter et al. [52] found that immediately after a single virtual gallery visit, several negative emotions (angry, irritable, anxious, tense, and unhappy) decreased in intensity and several positive emotions (relaxed, awe, moved, and chills) increased in intensity. Single visits to art museums have been associated with immediate reductions in cortisol [35,36], lowered blood pressure [108], and increased subjective well-being [36] and feelings of social connection [40,109]. A recent study concluded that various impacts can be detected immediately after non-virtual art engagement, but some of these are not long-lasting [110]. To address this limitation, future research should include detailed assessment of flourishing across multiple time points in addition to overall pre and post flourishing assessments.

***Viewing was brief.*** Participants only spent 15 min in each session. This amount of time may well have been too short to result in growth of flourishing. Many of the studies documenting flourishing benefits of engagement with visual art have focused on entire museum visits or long-term behavioral patterns of art museum visitation (see [34] for review), both of which are substantially longer than the 15 min viewing periods used in the present research. Although a strength of the present research was the repeated viewing sessions, it did constrain the length of each individual session. Future research should address this limitation through varying visit times, including visit times longer than 15 min, to determine whether length of visit plays a role in the flourishing impacts of virtual art engagement. Additionally, future research should also allow for participant-determined visit lengths, as some initial work [111] indicates that it is the subjective visit length (e.g., too short, too long, or ideal) that may be more important than the objective visit length.

***Viewing was virtual.*** Finally, another reason for our lack of effects could be due to the fact that the viewing was virtual. We badly need studies directly comparing effects of flourishing on virtual vs. museum viewing experiences to understand the similarities and differences between these forms of engagement. This is especially important as the interest in and development of virtual art experiences is growing [49,50,51].

## 5. Conclusions

The present research examined the effects of repeated visitation to a virtual art gallery on several global flourishing measures and the role of immersion in these effects. This project is the first to examine how repeated virtual art engagement may benefit flourishing. Although the present findings did not show shifts in global flourishing metrics, this study did suggest directions for future research, including the using more state-like measures of flourishing, rather than general or global measures, measuring flourishing immediately following the art experiences, increasing the length of time in the galleries, and comparing effects of virtual vs. non-virtual art viewing. Additionally, studying the flourishing effects of visual art in a virtual context may further allow us to identify qualities of specific artworks (e.g., brightness, content, style) that are more conducive to flourishing or are better able to provide immersive experiences. Given their ease of access and the increasing number of virtual art galleries, understanding these experiences and how they may impact our flourishing deserves greater attention.

## Figures and Tables

**Table 1:** Demographic Information.

	Screening (*n* = 2000)	Invited (*n* = 1479)	Final Sample (*n* = 687)
Age	*M* = 44.11, *SD* = 15.87 *Range =* 18–84	*M* = 41.87, *SD* = 15.35, *Range =* 18–81	*M* = 47.31, *SD* = 14.96, *Range* = 18–79
Race	American Indian = 1.75% Asian = 7.60% Black or African American = 13.04% Hispanic, Latino, or Spanish origin = 5.50% Middle Eastern or North African = 0.75% Native Hawaiian or Pacific Islander = 0.10% White = 76.51% Other = 0.90%	American Indian = 1.96% Asian = 7.57% Black or African American = 11.97% Hispanic, Latino, or Spanish origin = 6.29% Middle Eastern or North African = 0.81% Native Hawaiian or Pacific Islander = 0.07% White = 77.42% Other = 0.74%	American Indian = 1.60% Asian = 6.70% Black or African American = 11.79% Hispanic, Latino, or Spanish origin = 4.51% Middle Eastern or North African = 0.87% Native Hawaiian or Pacific Islander = 0.00% White = 80.06% Other = 0.73%
Gender	Female = 50.07% Male = 47.73%, Other = 2.20%	Female = 46.18% Male = 51.12% Other = 2.70%	Female = 50.85% Male = 47.60% Other = 1.46%
Education	Did not finish HS = 0.50% HS = 10.09% College, no degree = 20.49% Associates = 9.65% Bachelors = 38.53% Masters = 16.39% Doctoral/Professional = 4.20%	Did not finish HS = 0.68% HS = 10.68% College, no degree = 21.77% Associates = 10.01% Bachelors = 37.12% Masters = 15.48% Doctoral/Professional = 4.19%	Did not finish HS = 0.00% HS = 9.61% College, no degree = 20.38% Associates = 9.46% Bachelors = 36.39% Masters = 19.36% Doctoral/Professional = 4.80%

**Table 2:** Experimental Conditions.

Condition	Instructions
No Slow Looking + No Framing *n* = 89	When you enter the gallery, take a moment to become familiar with the navigation tools. While in the gallery, spend your time looking at whichever artworks you would like. Please continue looking at art in the gallery for the full 15 min. The survey will automatically advance once the viewing period is finished.
Slow Looking + No Framing *n* = 63	For the first 5 min of your visit, take a moment to become familiar with the navigation tools, look around the rooms, and select a single artwork you would like to look at more closely. Adjust your view of this work of art so that you are close to the work and see only this artwork. When you are close to the artwork you’ve selected, click your mouse to enlarge the artwork. Spend the next 10 min looking at this work of art. The survey will automatically advance once the viewing period is finished.
No Slow Looking + Mindful Framing *n* = 94	As you view the art, engage in mindful looking. Focus your awareness on the moment—notice your emotions, how you are feeling, and the thoughts that are passing through your mind. Let the outside world go, and be present in the moment. When you enter the gallery and view the art, take note of your reactions to it. Do your emotions change? What thoughts cross your mind? Just notice, without judgement, what you are seeing, feeling, and thinking.
No Slow Looking + Curious Framing *n* = 85	As you view the art, engage in curious looking. Notice the details of the art. What colors and lines do you see? What questions do you have about the art? How do you interpret what is being depicted in the art? Why do you interpret it in that way? Is there anything surprising about what you see? Why is it surprising? Be curious about what you are seeing.
No Slow Looking + Social Framing *n* = 84	As you view the art, consider how the art connects to important relationships in your life. You may even choose to focus on one specific relationship. Which relationship do you think of as you look at the art? Why does the art resonate with this relationship? Does the art call up certain emotions or thoughts about your relationship? Consider as vividly as you can how the art connects with your relationship.
Slow Looking + Mindful Framing *n* = 63	For the first 5 min of your visit, take a moment to become familiar with the navigation tools, look around the rooms, and select a single artwork you would like to look at more closely. Adjust your view of this work of art so that you are close to the work and see only this artwork. When you are close to the artwork you’ve selected, click your mouse to enlarge the artwork. Spend the next 10 min looking at this work of art. As you view the art, engage in mindful looking. Focus your awareness on the moment—notice your emotions, how you are feeling, and the thoughts that are passing through your mind. Let the outside world go, and be present in the moment. When you enter the gallery and view the art, take note of your reactions to it. Do your emotions change? What thoughts cross your mind? Just notice, without judgement, what you are seeing, feeling, and thinking. The survey will automatically advance once the viewing period is finished.
Slow Looking + Curious Framing *n* = 55	For the first 5 min of your visit, take a moment to become familiar with the navigation tools, look around the rooms, and select a single artwork you would like to look at more closely. Adjust your view of this work of art so that you are close to the work and see only this artwork. When you are close to the artwork you’ve selected, click your mouse to enlarge the artwork. Spend the next 10 min looking at this work of art. As you view the art, engage in curious looking. Notice the details of the art. What colors and lines do you see? What questions do you have about the art? How do you interpret what is being depicted in the art? Why do you interpret it in that way? Is there anything surprising about what you see? Why is it surprising? Be curious about what you are seeing. The survey will automatically advance once the viewing period is finished.
Slow Looking + Social Framing *n* = 54	For the first 5 min of your visit, take a moment to become familiar with the navigation tools, look around the rooms, and select a single artwork you would like to look at more closely. Adjust your view of this work of art so that you are close to the work and see only this artwork. When you are close to the artwork you’ve selected, click your mouse to enlarge the artwork. Spend the next 10 min looking at this work of art. As you view the art, consider how the art connects to important relationships in your life. You may even choose to focus on one specific relationship. Which relationship do you think of as you look at the art? Why does the art resonate with this relationship? Does the art call up certain emotions or thoughts about your relationship? Consider as vividly as you can how the art connects with your relationship. The survey will automatically advance once the viewing period is finished.
Non-Gallery Reading Control *n* = 100	*Readings focused on the topics of abstract expressionism, gothic art, impressionism, and rococo. Readings did not contain any art images. To view the narratives please see:* https://osf.io/r7pgy/

**Table 3:** Experimental condition predicting immersion.

	Estimate (*b*)	SE	*p*
Gallery vs. Non-Gallery	0.66	0.13	<0.001
Slow Looking vs. No Slow Looking	0.06	0.10	0.582
Framing vs. No Framing	0.17	0.11	0.129
Slow Looking × Framing			
Slow Looking	−0.17	0.20	0.395
Framing	0.05	0.15	0.739
Interaction	0.31	0.23	0.182

## Data Availability

Data and study materials are available on the Open Science Framework at: https://osf.io/r7pgy/. Code and additional information for the Open Gallery for Arts Research is available at: https://github.com/HHF-Penn/OGAR.

## Supplementary Materials

The following supporting information can be downloaded at: https://www.mdpi.com/article/10.3390/bs12120500/s1. Table S1: Details for artworks in the virtual galleries; Table S2: Descriptive statistics for the well-being variables; Table S3: Correlations among well-being variables pre-intervention (upper diagonal) and post-intervention (lower diagonal); Table S4: Gallery (1) vs Non-gallery (0) regression predicting well-being; Table S5: Gallery (1) vs non-gallery (0) and interaction between condition and pre-test flourishing predicting post-test flourishing; Table S6: Slow looking (1) vs. No slow looking (0) predicting well-being; Table S7: Slow Looking (1) vs. no slow looking (0) and the condition and pre-test flourishing interaction predicting post-test flourishing; Table S8: Immersive mindset framing (1) vs. No framing (0) predicting well-being; Table S9: Immersive mindset framing (1) vs no framing (0) and interaction between condition and pretest flourishing predicting post-test flourishing.; Table S10: Slow looking, Immersive Mindset Framing, and Interaction predicting well-being; Table S11: All condition factors and interactions predicting post-test flourishing; Table S12: Latent immersion factor predicting well-being; Table S13: Latent art topic factor predicting well-being; Table S14: Latent feeling topic factor predicting well-being; Figure S1: Changes in well-being indices before and after the intervention; Figure S2: Interactions between experimental condition and pre-test flourishing predicting post-test flourishing.
