# Supplementary material for: Examining the Flourishing Impacts of Repeated Visits to a Virtual Art Museum and the Role of Immersion
Source: Behav Sci (Basel). 2022 Dec 7;12(12):500. doi: 10.3390/bs12120500 (PMC9774448; doi:10.3390/bs12120500)
Supplement: Supplementary file 1 [file behavsci-12-00500-s001.zip › behavsci-2020428-supplementary.pdf]

Supplementary Materials

Supplementary Table S1.

Details for artworks in the virtual galleries

Title	Date	Artist
Technical Screening Gallery		
Dances at the Spring	1912	Francis Picabia
Dog Barking at the Moon	1926	Joan Miro
Fantasia No. 5	1973	Maya Schock
Old Woman Reading	1621-1623	Jan Lievens
Saint Thomas Receiving the Virgin's Belt with Saints Michael Archangel, Augustine, Margaret of Antioch, and Catherine of Alexandria	1467	Neri di Bicci
Soldiers beside a Fireplace	1628-1632	Willem Cornelisz
Still Life with a Tortoise	1743	Possibly by Thomas Black
Tsakali of Five-Fold Throne of the Tathagatha	18th - 19th century	Artist/maker unknown, Tibetan
Vriksha (Tree) Pichhwai	18th century	Artist/maker unknown, Indian
Xanadu	1972	Murray Dessner
Week 1 Gallery		
A Critic and his Artist Friends (Portrait of Christian Brinton and Nine Artists)	1924	David Davidovich Burliuk
A Gift Not Given	1981	Alexandria Lerner
Abstraction	1939-1944	Ad Reinhardt
Bedroom Painting No. 7	1967-1969	Tom Wesselmann
Bellowing Bull	17th century	Copy after Paulus Potter
Camouflage Self-Portrait	1986	Andy Warhol
Composition	1940	Suzy Frelinghuysen (Estelle Condit Frelinghuysen)
Coppers	1960	Juan Mingorance
Cow and Calf	1929	George Biddle
England	Date unknown	Ellis Ruley
Garbo Dance Around the Tree of Life	18th century	Artist/maker unknown, Indian
Grass	2000	Howard Hodgkin

Jain Cosmogram	1700	Artist/maker unknown, Indian
John Fitzgerald Kennedy	1962-1963	Sergio Lombardo
Lotus	1960	Guo Dawei (David Kwo)
Maharao Ratan of Bundi Attop His Horse	1680	Artist/maker unknown, Indian
Maine Landscape No. 27	1909	Marsden Hartley
Mountains	1910-1911	Marguerite Thompson Zorach
Ocean Liner in New York	Date unknown	Justin McCarthy
Pekingese	1909	Sir Alfred Munnings
Private Aquarium	1959	Abraham P. Hankins
Still Life with Apples and a Glass of Wine	1877-1879	Paul Cezanne
Still Life: Mountain Dew	1827	Chester Harding
Study for "Franklin's Footpath"	1971	Gene Davis
Thakur Balwant Singh Presides at a Dance Performance	1890	Attributed to Mohan Lal, Indian
The Birth of Venus	1635 or 1636	Nicolas Poussin
The Ocean Seen from the Corridor	2006	Trevor Winkfield
Untitled	1955-1986	Emery Blagdon
Untitled (Bird Arabesque)	1933	Rabindranath Tagore
Whoopee at Sloppy Jo's	1933	George Biddle
Week 2 Gallery		
A Bum Bock	1857	Artist/maker unknown
Album Paintings of Household Gods: the Vanguard with White Horse (Baima xianfeng)	1900	Zhou Peichun
Animal Caught in a Trap	1929	Andre Masson
Beth	1960	Morris Louis
Bull Fight	1985	William L. Hawkins
Christ Healing a Lunatic and Judas Receiving Thirty Pieces of Silver	1425-1426	Francesco d'Antonio
Compost with Bean Pod	1998-2004	Emily Brown
Elijah in the Desert	1834	Moritz Berendt
Fight Between a Man and a Tiger	20th century	Artist/maker unknown, Indian
Girl with a Mask	1945	Juan Soriano
Horse, Pipe, and Red Flower	1920	Joan Miro
Hound Coursing a Stag	1762	George Stubbs

Improvisation No. 29 (The Swan)	1912	Vasily Kandinsky
Krishna Shares Food with Balarama and the Cowherds during the Rainy Season	1570-1575	Artist/maker unknown, Indian
Night Sea	1977	Edna Andrade
Nomad with a Tribute Horse	19th century	Artist/maker unknown, Chinese
Painting	1933	Joan Miro
Portrait of Marian and Betty	1970	Beauford Delaney
Purple Lips	1912	Alexey von Jawlensky
Rift	5-Jun	Odili Donald Odita
Scene in a Tavern	17th century	Adriaen Brouwer
Still Life	1850	Artist/maker unknown, American
Still Life with Peaches	1927	Luigi Lucioni
Still Life with Roses of Dijon	1882	Ignace-Henri-Jean-Theodore Fantin-Latour
Summer Landscape	1964	Roland Conrad Petersen
Taboo	1963	Jacob Lawrence
The City	1919	Fernand Leger
Three Nudes (The Aunts)	1930	Julio Castellanos
Truth Rescued from Romance	1980	Katherine Porter
Wood Interior	1914	Man Ray
Week 3 Gallery		
A Rooster and a Hen Fighting	17th century	Follower of Melchior de Hondecoeter
Album Paintings of Household Gods: God of Happiness	1900	Zhou Peichun
Aux Enfants	1960	Abraham P. Hankins
Bad Reception	1976	William Schwedler
Boats, Gloucester	1917	Stuart Davis
Brown Shapes White	1941	Alice Trumbull Mason
Bullfight	1934	Pablo Ruiz y Picasso
Cultivated Pursuits	Late 19th century	Artist/maker unknown, Chinese
Daily Domestic Arguments	1913	Leonardo Dudreville
Gayety Burlesk	1932	Reginald Marsh
Hammer	1981	Peter Nadin
Landscape	1670	Liu Du
Little Painting with Yellow (Improvisation)	1914	Vasily Kandinsky
Nude Reclining by the Sea	1868	Gustave Courbet
Pertaining to Yachts and Yachting	1922	Charles Sheeler
Portrait of the Widow Marion Knapp [nÃ©e Graham, later Baroness Bateman]	1903	Franz von Lenbach

Reclining Nude	1976	Tommy Dale Palmore
Self-Portrait	1975	Martha Mayer Erlebacher
Stream in Winter	1940	Walter Elmer Schofield
Study of Three Heads	1870	Henri Regnault
The Bandaged Night	1956	William Scharf
The Battle of the USS "Kearsarge" and the CSS "Alabama"	1864	Edouard Manet
The Departure	1951	Grandma Moses (Anna Mary Robertson)
The Dessert	1877-1879	Paul Cezanne
The Emblem of Christ Appearing to Constantine	1622	Peter Paul Rubens
The Fatal Temple	1914	Giorgio de Chirico
The Hermitage	1924	Joan Miro
Trying to find our spot off in that light, light off in that spot	2014	Jayson Musson
View	2000	John Moore
Wounded Stag	19th century	Imitator of Gustave Courbet
Week 4 Gallery		
Album Paintings of Household Gods: Heavenly King Virupaksa	1900	Zhou Peichun
Cockfight	1930	Andre Masson
Composition	1933	Jean Helion
Don't Drink the Water	1968	David Pease
Egg Rocker	1952	Alan Davie
Elegy to the Spanish Republic	1958-1961	Robert Motherwell
Elephant in Battle	1750-1770	Artist/maker unknown, Indian
Favorite Chestnut Hunter of Lady Frances Pierrepont	1799	Benjamin Marshall
Girl Combing Her Hair	1910	Max Pechstein
Marriage Painting	20th century	Warli people, Indian
Mocking of Christ	1454	Joan Reixach
Musicians	Date unknown	Jon Serl
New Mexico Landscape	1919-1920	Marsden Hartley
Pennsylvania Landscape	1925	Charles Sheeler
Playing Card Depicting the Goddess Durga	1800-1825	Artist/maker unknown, Indian
Portrait of John Hart	1798	Charles Peale Polk
Present Futures	2006	Moe Brooker

Rock and Anchor	1961	Morris Atkinson Blackburn
Still Life: Cyclamen	1925	Vera M. White
The Beach, Newport (In the Sand)	1919	George Bellows
The Death of Sardanapalus	1844	Ferdinand-Victor-Eugene Delacroix
The Deposition	1961	Bob Thompson
The Lament of Aminta	1610-1615	Bartolomeo Cavarozzi
The Nativity and the Arrival of the Magi	Late 15th century	Artist/maker unknown, active Crete, Greek
The Orchard Window	1918	Daniel Garber
The passage of the bride II	2004	Richard Hamilton
Untitled Painting #10	1963	Robert Ryman
Untitled XXI	1982	Attributed to Willem de Kooning
View of the Yare	19th century	Artist/maker unknown, English
Winter Landscape	Late 1660s	Jacob Isaacksz. van Ruisdael

Supplementary Table S2.

Descriptive statistics for the well-being variables

	Pre-Intervention			Post-Intervention		
**Variable**	**Mean**	**SD**	**Cronbach's α**	**Mean**	**SD**	**Cronbach's α**
Support	4.13	0.76	0.86	4.17	0.76	0.87
Community	3.23	0.95	0.76	3.20	0.95	0.79
Trust	3.37	0.84	0.84	3.37	0.87	0.86
Respect	3.73	0.78	0.81	3.75	0.79	0.82
Loneliness	2.17	1.03	0.82	2.16	1.06	0.85
Belonging	3.25	0.96	0.85	3.28	0.99	0.88
Engagement	3.91	0.67	0.69	3.92	0.68	0.71
Skills	3.52	0.93	0.86	3.51	0.93	0.87
Learning	3.81	0.85	0.75	3.87	0.84	0.78
Accomplishment	3.22	1.01	0.92	3.22	1.04	0.93
Self-Efficacy	3.94	0.79	0.82	3.93	0.83	0.84
Self-Worth	3.59	0.93	0.85	3.61	0.95	0.88
Control	4.10	0.84	0.83	4.06	0.88	0.85
Meaning	3.57	1.05	0.91	3.58	1.06	0.92
Optimism	3.61	1.00	0.90	3.61	0.98	0.90
Life Satisfaction	3.25	1.06	0.92	3.24	1.06	0.92
Positive Feelings	3.53	1.02	0.94	3.49	1.03	0.95
Negative Feelings	2.04	1.08	0.93	2.09	1.10	0.93
Overall Thriving	3.89	0.84	0.97	3.86	0.86	0.98
Stress	1.85	0.84	0.90	1.84	0.86	0.91
Depression	1.77	0.96	0.95	1.76	0.97	0.95
Anxiety	1.44	0.66	0.86	1.45	0.68	0.88
Relatedness Satisfaction	3.60	0.97	0.88	3.59	1.01	0.89
Relatedness Dissatisfaction	2.24	0.99	0.70	2.19	1.00	0.74
Autonomy Satisfaction	3.65	0.78	0.75	3.65	0.80	0.79
Autonomy Dissatisfaction	2.32	0.93	0.72	2.41	0.97	0.74
Competency Satisfaction	3.35	0.86	0.81	3.37	0.87	0.83
Competency Dissatisfaction	2.31	1.04	0.81	2.28	1.02	0.81
Relatedness Overall	1.36	1.69	0.82	1.41	1.79	0.85
Autonomy Overall	1.32	1.50	0.79	1.25	1.58	0.83
Competency Overall	1.04	1.57	0.79	1.10	1.58	0.81

Supplementary Table S3.

Correlations among well-being variables pre-intervention (upper diagonal) and post-intervention (lower diagonal)

	1	2	3	4	5	6	7	8	9	10
1. Support	---	0.30	0.44	0.57	-0.65	0.50	0.33	0.41	0.25	0.41
2. Community	0.36	---	0.39	0.39	-0.32	0.54	0.30	0.43	0.19	0.41
3. Trust	0.44	0.45	---	0.49	-0.40	0.57	0.30	0.34	0.20	0.41
4. Respect	0.61	0.44	0.53	---	-0.55	0.56	0.37	0.54	0.20	0.51
5. Loneliness	-0.66	-0.41	-0.38	-0.56	---	-0.54	-0.29	-0.44	-0.18	-0.53
6. Belonging	0.48	0.60	0.55	0.60	-0.55	---	0.34	0.47	0.23	0.54
7. Engagement	0.38	0.37	0.31	0.41	-0.34	0.34	---	0.47	0.37	0.40
8. Skills	0.40	0.45	0.37	0.54	-0.44	0.48	0.49	---	0.33	0.66
9. Learning	0.33	0.25	0.27	0.33	-0.25	0.25	0.36	0.30	---	0.22
10. Accomplishment	0.45	0.47	0.41	0.57	-0.55	0.54	0.40	0.68	0.30	---
11. Self-Efficacy	0.51	0.36	0.39	0.58	-0.46	0.47	0.48	0.59	0.37	0.62
12. Self-Worth	0.49	0.48	0.35	0.58	-0.50	0.53	0.45	0.69	0.30	0.64
13. Control	0.39	0.23	0.26	0.41	-0.44	0.32	0.30	0.37	0.18	0.42
14. Meaning	0.49	0.44	0.35	0.57	-0.55	0.56	0.41	0.61	0.31	0.72
15. Optimism	0.52	0.43	0.48	0.60	-0.57	0.57	0.44	0.60	0.37	0.76
16. Life Satisfaction	0.47	0.47	0.41	0.54	-0.61	0.54	0.37	0.64	0.28	0.82
17. Positive Feelings	0.53	0.48	0.48	0.61	-0.66	0.58	0.42	0.59	0.31	0.73
18. Negative Feelings	-0.52	-0.43	-0.45	-0.60	0.67	-0.56	-0.36	-0.54	-0.27	-0.64
19. Overall Thriving	0.73	0.47	0.50	0.67	-0.75	0.60	0.43	0.58	0.33	0.70
20. Stress	-0.34	-0.31	-0.35	-0.41	0.51	-0.38	-0.19	-0.35	-0.14	-0.38
21. Depression	-0.51	-0.39	-0.40	-0.57	0.65	-0.51	-0.35	-0.50	-0.23	-0.60
22. Anxiety	-0.33	-0.22	-0.26	-0.34	0.44	-0.29	-0.18	-0.29	-0.11	-0.35
23. Relatedness Satisfaction	0.71	0.44	0.44	0.57	-0.71	0.53	0.36	0.44	0.30	0.53
24. Relatedness Dissatisfaction	-0.50	-0.34	-0.36	-0.51	0.75	-0.44	-0.26	-0.38	-0.19	-0.45
25. Autonomy Satisfaction	0.50	0.37	0.44	0.59	-0.51	0.48	0.48	0.62	0.30	0.60
26. Autonomy Dissatisfaction	-0.36	-0.24	-0.33	-0.45	0.48	-0.35	-0.27	-0.36	-0.17	-0.41

27. Competency Satisfaction	0.36	0.40	0.34	0.47	-0.38	0.40	0.45	0.66	0.31	0.61
28. Competency Dissatisfaction	-0.39	-0.30	-0.33	-0.48	0.50	-0.38	-0.24	-0.40	-0.19	-0.46
29. Relatedness Overall	0.68	0.44	0.45	0.60	-0.82	0.55	0.35	0.46	0.28	0.55
30. Autonomy Overall	0.47	0.34	0.42	0.57	-0.55	0.46	0.41	0.54	0.25	0.56
31. Competency Overall	0.45	0.42	0.40	0.57	-0.54	0.47	0.41	0.62	0.29	0.63

	11	12	13	14	15	16	17	18	19	20
1. Support	0.45	0.44	0.35	0.47	0.46	0.45	0.48	-0.48	0.70	-0.36
2. Community	0.33	0.49	0.17	0.39	0.37	0.41	0.41	-0.34	0.39	-0.26
3. Trust	0.35	0.37	0.25	0.37	0.49	0.43	0.53	-0.46	0.53	-0.38
4. Respect	0.54	0.54	0.43	0.54	0.55	0.53	0.60	-0.53	0.62	-0.43
5. Loneliness	-0.44	-0.45	-0.43	-0.57	-0.53	-0.59	-0.63	0.65	-0.73	0.49
6. Belonging	0.46	0.53	0.31	0.55	0.58	0.54	0.60	-0.56	0.63	-0.45
7. Engagement	0.45	0.44	0.22	0.40	0.40	0.37	0.40	-0.31	0.36	-0.10
8. Skills	0.59	0.68	0.37	0.57	0.56	0.62	0.59	-0.50	0.56	-0.30
9. Learning	0.29	0.27	0.10	0.25	0.27	0.20	0.23	-0.20	0.24	-0.12
10. Accomplishment	0.61	0.67	0.37	0.70	0.74	0.82	0.69	-0.62	0.66	-0.44
11. Self-Efficacy	---	0.59	0.39	0.58	0.65	0.56	0.61	-0.53	0.59	-0.35
12. Self-Worth	0.60	---	0.28	0.69	0.63	0.61	0.60	-0.52	0.57	-0.31
13. Control	0.45	0.31	---	0.37	0.39	0.37	0.41	-0.48	0.48	-0.36
14. Meaning	0.61	0.71	0.40	---	0.68	0.69	0.66	-0.62	0.66	-0.42
15. Optimism	0.68	0.62	0.45	0.70	---	0.73	0.77	-0.69	0.73	-0.50
16. Life Satisfaction	0.57	0.61	0.42	0.69	0.75	---	0.74	-0.66	0.71	-0.44
17. Positive Feelings	0.61	0.59	0.41	0.67	0.79	0.76	---	-0.81	0.88	-0.60
18. Negative Feelings	-0.56	-0.57	-0.45	-0.65	-0.73	-0.69	-0.82	---	-0.91	0.64
19. Overall Thriving	0.63	0.61	0.48	0.68	0.76	0.74	0.89	-0.92	---	-0.63
20. Stress	-0.36	-0.35	-0.34	-0.38	-0.46	-0.45	-0.58	0.66	-0.61	---
21. Depression	-0.57	-0.56	-0.45	-0.62	-0.68	-0.65	-0.74	0.83	-0.80	0.74
22. Anxiety	-0.34	-0.33	-0.30	-0.32	-0.40	-0.38	-0.47	0.57	-0.53	0.73
23. Relatedness Satisfaction	0.49	0.52	0.38	0.54	0.55	0.57	0.62	-0.56	0.70	-0.44

24. Relatedness Dissatisfaction	-0.40	-0.40	-0.43	-0.43	-0.50	-0.53	-0.58	0.62	-0.64	0.60
25. Autonomy Satisfaction	0.57	0.53	0.51	0.57	0.58	0.60	0.62	-0.59	0.65	-0.46
26. Autonomy Dissatisfaction	-0.38	-0.29	-0.55	-0.37	-0.44	-0.48	-0.52	0.54	-0.56	0.58
27. Competency Satisfaction	0.58	0.63	0.32	0.54	0.56	0.53	0.56	-0.50	0.53	-0.30
28. Competency Dissatisfaction	-0.48	-0.42	-0.40	-0.46	-0.47	-0.47	-0.53	0.60	-0.59	0.61
29. Relatedness Overall	0.51	0.51	0.46	0.55	0.59	0.62	0.67	-0.67	0.75	-0.58
30. Autonomy Overall	0.52	0.45	0.60	0.52	0.57	0.60	0.63	-0.64	0.67	-0.59
31. Competency Overall	0.63	0.61	0.43	0.59	0.61	0.60	0.65	-0.66	0.68	-0.56

	21	22	23	24	25	26	27	28	29	30	31
1. Support	-0.51	-0.31	0.68	-0.49	0.47	-0.32	0.33	-0.38	0.67	0.45	0.43
2. Community	-0.32	-0.16	0.35	-0.21	0.29	-0.17	0.35	-0.26	0.32	0.26	0.37
3. Trust	-0.41	-0.25	0.35	-0.36	0.35	-0.31	0.31	-0.35	0.41	0.38	0.41
4. Respect	-0.58	-0.37	0.50	-0.45	0.52	-0.38	0.47	-0.47	0.55	0.51	0.57
5. Loneliness	0.65	0.42	-0.68	0.71	-0.46	0.43	-0.38	0.53	-0.81	-0.51	-0.56
6. Belonging	-0.56	-0.34	0.47	-0.45	0.44	-0.34	0.42	-0.44	0.53	0.44	0.52
7. Engagement	-0.32	-0.12	0.37	-0.25	0.44	-0.21	0.45	-0.24	0.36	0.36	0.40
8. Skills	-0.50	-0.26	0.44	-0.36	0.60	-0.32	0.63	-0.39	0.47	0.51	0.60
9. Learning	-0.19	-0.10	0.24	-0.15	0.24	-0.08	0.31	-0.11	0.22	0.18	0.24
10. Accomplishment	-0.63	-0.38	0.50	-0.46	0.56	-0.37	0.58	-0.46	0.56	0.52	0.62
11. Self-Efficacy	-0.57	-0.36	0.45	-0.35	0.54	-0.28	0.57	-0.47	0.46	0.45	0.62
12. Self-Worth	-0.54	-0.27	0.47	-0.34	0.47	-0.21	0.55	-0.37	0.47	0.38	0.55
13. Control	-0.43	-0.30	0.34	-0.39	0.47	-0.53	0.29	-0.39	0.42	0.58	0.42
14. Meaning	-0.65	-0.36	0.51	-0.47	0.52	-0.35	0.52	-0.47	0.57	0.50	0.60
15. Optimism	-0.70	-0.41	0.50	-0.45	0.55	-0.39	0.51	-0.48	0.55	0.53	0.59
16. Life Satisfaction	-0.68	-0.38	0.55	-0.49	0.55	-0.42	0.52	-0.47	0.60	0.55	0.60
17. Positive Feelings	-0.78	-0.48	0.54	-0.55	0.58	-0.45	0.53	-0.56	0.63	0.59	0.66
18. Negative Feelings	0.81	0.54	-0.48	0.59	-0.51	0.50	-0.46	0.61	-0.62	-0.58	-0.66
19. Overall Thriving	-0.81	-0.53	0.61	-0.63	0.59	-0.52	0.50	-0.61	0.72	0.63	0.68
20. Stress	0.74	0.74	-0.36	0.56	-0.47	0.52	-0.30	0.62	-0.54	-0.57	-0.58

21. Depression	---	0.67	-0.53	0.60	-0.56	0.48	-0.52	0.65	-0.66	-0.59	-0.72
22. Anxiety	0.68	---	-0.29	0.49	-0.37	0.38	-0.30	0.54	-0.45	-0.43	-0.53
23. Relatedness Satisfaction	-0.57	-0.36	---	-0.48	0.53	-0.32	0.46	-0.38	0.86	0.48	0.51
24. Relatedness Dissatisfaction	0.62	0.45	-0.58	---	-0.49	0.64	-0.25	0.66	-0.86	-0.66	-0.57
25. Autonomy Satisfaction	-0.59	-0.39	0.61	-0.50	---	-0.52	0.53	-0.47	0.59	0.85	0.61
26. Autonomy Dissatisfaction	0.54	0.43	-0.43	0.61	-0.59	---	-0.17	0.60	-0.56	-0.89	-0.49
27. Competency Satisfaction	-0.49	-0.28	0.47	-0.34	0.56	-0.23	---	-0.35	0.41	0.39	0.78
28. Competency Dissatisfaction	0.64	0.53	-0.43	0.61	-0.49	0.60	-0.39	---	-0.60	-0.62	-0.86
29. Relatedness Overall	-0.66	-0.46	0.89	-0.89	0.63	-0.58	0.46	-0.59	---	0.66	0.63
30. Autonomy Overall	-0.63	-0.46	0.57	-0.63	0.87	-0.91	0.42	-0.62	0.67	---	0.62
31. Competency Overall	-0.68	-0.50	0.54	-0.58	0.62	-0.51	0.81	-0.86	0.63	0.63	---

Supplementary Table S4.

Gallery (1) vs Non-gallery (0) regression predicting well-being

**Variable**	**Predictor**	***Estimate***	***SE***	***p***	***f²***
Support	Pre	0.79***	0.02	< 0.001	1.79
	Gallery vs. Non-Gallery	0.06	0.06	0.336	0.00
Community	Pre	0.78***	0.02	< 0.001	1.52
	Gallery vs. Non-Gallery	0.09	0.07	0.185	0.00
Trust	Pre	0.82***	0.02	< 0.001	1.63
	Gallery vs. Non-Gallery	0.05	0.07	0.512	0.00
Respect	Pre	0.79***	0.02	< 0.001	1.62
	Gallery vs. Non-Gallery	0.07	0.07	0.33	0.00
Loneliness	Pre	0.87***	0.02	< 0.001	2.45
	Gallery vs. Non-Gallery	-0.09	0.06	0.134	0.00
Belonging	Pre	0.83***	0.02	< 0.001	1.80
	Gallery vs. Non-Gallery	0.05	0.07	0.443	0.00
Engagement	Pre	0.67***	0.03	< 0.001	0.80
	Gallery vs. Non-Gallery	0.16	0.08	0.051	0.01
Skills	Pre	0.76***	0.03	< 0.001	1.33
	Gallery vs. Non-Gallery	-0.07	0.07	0.295	0.00
Learning	Pre	0.61***	0.03	< 0.001	0.63
	Gallery vs. Non-Gallery	0.00	0.08	0.966	0.00
Accomplishment	Pre	0.88***	0.02	< 0.001	2.86
	Gallery vs. Non-Gallery	0.01	0.06	0.859	0.00
Self-Efficacy	Pre	0.84***	0.02	< 0.001	1.91
	Gallery vs. Non-Gallery	0.00	0.07	0.992	0.00
Self-Worth	Pre	0.80***	0.02	< 0.001	1.61
	Gallery vs. Non-Gallery	0.12	0.07	0.091	0.00
Control	Pre	0.70***	0.03	< 0.001	0.80
	Gallery vs. Non-Gallery	0.15	0.09	0.07	0.00
Meaning	Pre	0.86***	0.02	< 0.001	2.62
	Gallery vs. Non-Gallery	0.11*	0.06	0.048	0.01
Optimism	Pre	0.84***	0.02	< 0.001	2.82
	Gallery vs. Non-Gallery	0.02	0.05	0.745	0.00
Life Satisfaction	Pre	0.88***	0.02	< 0.001	3.58
	Gallery vs. Non-Gallery	-0.08	0.05	0.101	0.00
Positive Feelings	Pre	0.86***	0.02	< 0.001	2.71
	Gallery vs. Non-Gallery	0.03	0.06	0.542	0.00
Negative Feelings	Pre	0.85***	0.02	< 0.001	2.28
	Gallery vs. Non-Gallery	0.01	0.06	0.933	0.00
Overall Thriving	Pre	0.87***	0.02	< 0.001	2.78
	Gallery vs. Non-Gallery	0.02	0.06	0.758	0.00
Stress	Pre	0.82***	0.02	< 0.001	1.81

	Gallery vs. Non-Gallery	0.03	0.07	0.658	0.00
Depression	Pre	0.85***	0.02	< 0.001	2.51
	Gallery vs. Non-Gallery	-0.03	0.06	0.618	0.00
Anxiety	Pre	0.86***	0.02	< 0.001	2.06
	Gallery vs. Non-Gallery	0.04	0.06	0.554	0.00
Relatedness Satisfaction	Pre	0.78***	0.03	< 0.001	1.26
	Gallery vs. Non-Gallery	-0.11	0.07	0.14	0.00
Relatedness Dissatisfaction	Pre	0.71***	0.03	< 0.001	0.94
	Gallery vs. Non-Gallery	-0.05	0.08	0.558	0.00
Autonomy Satisfaction	Pre	0.68***	0.03	< 0.001	0.77
	Gallery vs. Non-Gallery	0.03	0.08	0.738	0.00
Autonomy Dissatisfaction	Pre	0.72***	0.03	< 0.001	0.92
	Gallery vs. Non-Gallery	-0.05	0.08	0.54	0.00
Competency Satisfaction	Pre	0.69***	0.03	< 0.001	0.89
	Gallery vs. Non-Gallery	-0.06	0.08	0.432	0.00
Competency Dissatisfaction	Pre	0.67***	0.03	< 0.001	0.89
	Gallery vs. Non-Gallery	0.08	0.08	0.326	0.00
Relatedness Overall	Pre	0.82***	0.03	< 0.001	1.51
	Gallery vs. Non-Gallery	-0.05	0.07	0.521	0.00
Autonomy Overall	Pre	0.79***	0.03	< 0.001	1.25
	Gallery vs. Non-Gallery	0.03	0.08	0.723	0.00
Competency Overall	Pre	0.77***	0.02	< 0.001	1.47

Note. Estimates represent unstandardized regression weights in pre-intervention standard deviation units. Effect size is Cohen's f^2 and can be interpreted as small = 0.02, medium = 0.15, large = 0.35.

Supplementary Table S5.

Gallery (1) vs non-gallery (0) and interaction between condition and pre-test flourishing predicting post-test flourishing.

**Variable**	**Predictor**	**Estimate**	***SE***	***p***	**Effect Size**
Support	Pre	0.78***	0.05	< 0.001	0.33
	Gallery	0.06	0.06	0.327	0.00
	Interaction	0.02	0.06	0.792	0.00
Community	Pre	0.71***	0.06	< 0.001	0.18
	Gallery	0.10	0.07	0.164	0.00
	Interaction	0.08	0.07	0.217	0.00
Trust	Pre	0.84***	0.06	< 0.001	0.25
	Gallery	0.04	0.07	0.555	0.00
	Interaction	-0.03	0.07	0.678	0.00
Respect	Pre	0.90***	0.06	< 0.001	0.35
	Gallery	0.06	0.07	0.416	0.00
	Interaction	-0.13*	0.06	0.046	0.00
Loneliness	Pre	0.98***	0.05	< 0.001	0.49
	Gallery	-0.09	0.06	0.128	0.00
	Interaction	-0.13*	0.06	0.024	0.00
Belonging	Pre	0.81***	0.06	< 0.001	0.25
	Gallery	0.05	0.07	0.421	0.00
	Interaction	0.03	0.07	0.675	0.00
Engagement	Pre	0.70***	0.07	< 0.001	0.14
	Gallery	0.16	0.08	0.055	0.01
	Interaction	-0.04	0.08	0.636	0.01
Skills	Pre	0.73***	0.07	< 0.001	0.17
	Gallery	-0.07	0.07	0.318	0.00
	Interaction	0.03	0.07	0.697	0.00
Learning	Pre	0.67***	0.07	< 0.001	0.12
	Gallery	0.00	0.08	0.993	0.00
	Interaction	-0.07	0.08	0.395	0.00
Accomplishment	Pre	0.96***	0.05	< 0.001	0.46
	Gallery	0.01	0.06	0.926	0.00
	Interaction	-0.09	0.06	0.11	0.00
Self-Efficacy	Pre	0.86***	0.06	< 0.001	0.30
	Gallery	0.00	0.07	0.997	0.00
	Interaction	-0.02	0.06	0.752	0.00
Self-Worth	Pre	0.94***	0.06	< 0.001	0.33
	Gallery	0.11	0.07	0.115	0.00
	Interaction	-0.16*	0.07	0.014	0.00
Control	Pre	0.80***	0.07	< 0.001	0.17
	Gallery	0.14	0.09	0.092	0.00

	Interaction	-0.11	0.08	0.165	0.00
Meaning	Pre	0.82***	0.05	< 0.001	0.38
	Gallery	0.12*	0.06	0.043	0.01
	Interaction	0.04	0.06	0.436	0.01
Optimism	Pre	0.85***	0.05	< 0.001	0.44
	Gallery	0.02	0.05	0.767	0.00
	Interaction	-0.02	0.05	0.754	0.00
Life Satisfaction	Pre	0.89***	0.05	< 0.001	0.50
	Gallery	-0.08	0.05	0.099	0.00
	Interaction	-0.01	0.05	0.843	0.00
Positive Feelings	Pre	0.88***	0.05	< 0.001	0.44
	Gallery	0.03	0.06	0.557	0.00
	Interaction	-0.02	0.06	0.736	0.00
Negative Feelings	Pre	0.86***	0.05	< 0.001	0.38
	Gallery	0.00	0.06	0.934	0.00
	Interaction	-0.02	0.06	0.714	0.00
Overall Thriving	Pre	0.92***	0.05	< 0.001	0.49
	Gallery	0.01	0.06	0.795	0.00
	Interaction	-0.06	0.05	0.245	0.00
Stress	Pre	0.83***	0.06	< 0.001	0.31
	Gallery	0.03	0.07	0.647	0.00
	Interaction	-0.02	0.06	0.776	0.00
Depression	Pre	0.97***	0.05	< 0.001	0.51
	Gallery	-0.02	0.06	0.7	0.00
	Interaction	-0.14*	0.06	0.014	0.00
Anxiety	Pre	0.98***	0.06	< 0.001	0.45
	Gallery	0.05	0.06	0.467	0.00
	Interaction	-0.14*	0.06	0.018	0.00
Relatedness Satisfaction	Pre	0.81***	0.07	< 0.001	0.21
	Gallery	-0.11	0.08	0.13	0.00
	Interaction	-0.04	0.07	0.629	0.00
Relatedness Dissatisfaction	Pre	0.73***	0.07	< 0.001	0.17
	Gallery	-0.04	0.08	0.573	0.00
	Interaction	-0.03	0.07	0.732	0.00
Autonomy Satisfaction	Pre	0.62***	0.08	< 0.001	0.10
	Gallery	0.04	0.08	0.671	0.00
	Interaction	0.07	0.08	0.414	0.00
Autonomy Dissatisfaction	Pre	0.66***	0.07	< 0.001	0.14
	Gallery	-0.06	0.08	0.461	0.00
	Interaction	0.08	0.07	0.308	0.00
Competency Satisfaction	Pre	0.65***	0.07	< 0.001	0.13
	Gallery	-0.06	0.08	0.449	0.00
	Interaction	0.05	0.08	0.493	0.00

Competency Dissatisfaction	Pre	0.60***	0.07	< 0.001	0.11
	Gallery	0.08	0.08	0.314	0.00
	Interaction	0.09	0.08	0.253	0.00
Relatedness Overall	Pre	0.82***	0.06	< 0.001	0.26
	Gallery	-0.05	0.07	0.521	0.00
	Interaction	0.00	0.07	0.974	0.00
Autonomy Overall	Pre	0.71***	0.06	< 0.001	0.18
	Gallery	0.04	0.08	0.598	0.00
	Interaction	0.09	0.07	0.192	0.00
Competency Overall	Pre	0.74***	0.06	< 0.001	0.21
	Gallery	-0.08	0.07	0.222	0.00
	Interaction	0.04	0.07	0.538	0.00

Note. Estimates represent unstandardized regression weights in pre-intervention standard deviation units. Effect size is Cohen's f^2 and can be interpreted as small = 0.02, medium = 0.15, large = 0.35.

Supplementary Table S6.

Slow looking (1) vs. No slow looking (0) predicting well-being

**Variable**	**Predictor**	**Estimate**	***SE***	***p***	***f*²**
Support	Pre	0.80***	0.02	< 0.001	1.79
	SL vs. No SL	0.03	0.05	0.528	0.00
Community	Pre	0.79***	0.03	< 0.001	1.62
	SL vs. No SL	-0.05	0.05	0.335	0.00
Trust	Pre	0.81***	0.03	< 0.001	1.65
	SL vs. No SL	0.04	0.05	0.398	0.00
Respect	Pre	0.77***	0.03	< 0.001	1.60
	SL vs. No SL	0.00	0.05	0.941	0.00
Loneliness	Pre	0.85***	0.02	< 0.001	2.36
	SL vs. No SL	0.03	0.05	0.554	0.00
Belonging	Pre	0.84***	0.03	< 0.001	1.83
	SL vs. No SL	0.01	0.05	0.895	0.00
Engagement	Pre	0.66***	0.03	< 0.001	0.82
	SL vs. No SL	0.01	0.06	0.925	0.00
Skills	Pre	0.76***	0.03	< 0.001	1.41
	SL vs. No SL	-0.08	0.05	0.122	0.00
Learning	Pre	0.60***	0.03	< 0.001	0.59
	SL vs. No SL	-0.04	0.07	0.557	0.00
Accomplishment	Pre	0.87***	0.02	< 0.001	2.88
	SL vs. No SL	0.10*	0.04	0.022	0.01
Self-Efficacy	Pre	0.84***	0.03	< 0.001	1.94
	SL vs. No SL	-0.02	0.05	0.637	0.00
Self-Worth	Pre	0.77***	0.03	< 0.001	1.50
	SL vs. No SL	-0.07	0.05	0.162	0.00
Control	Pre	0.69***	0.03	< 0.001	0.78
	SL vs. No SL	0.05	0.06	0.442	0.00
Meaning	Pre	0.87***	0.02	< 0.001	2.78
	SL vs. No SL	-0.06	0.04	0.183	0.00
Optimism	Pre	0.84***	0.02	< 0.001	2.80
	SL vs. No SL	0.01	0.04	0.884	0.00
Life Satisfaction	Pre	0.88***	0.02	< 0.001	3.67
	SL vs. No SL	0.02	0.04	0.602	0.00
Positive Feelings	Pre	0.86***	0.02	< 0.001	2.71
	SL vs. No SL	0.00	0.04	0.986	0.00
Negative Feelings	Pre	0.84***	0.02	< 0.001	2.22
	SL vs. No SL	0.00	0.05	0.988	0.00
Overall Thriving	Pre	0.86***	0.02	< 0.001	2.74
	SL vs. No SL	0.02	0.04	0.708	0.00
Stress	Pre	0.81***	0.03	< 0.001	1.77

	SL vs. No SL	0.03	0.05	0.550	0.00
Depression	Pre	0.83***	0.02	< 0.001	2.45
	SL vs. No SL	0.03	0.04	0.544	0.00
Anxiety	Pre	0.83***	0.03	< 0.001	1.78
	SL vs. No SL	0.00	0.05	0.944	0.00
Relatedness Satisfaction	Pre	0.77***	0.03	< 0.001	1.23
	SL vs. No SL	0.10	0.06	0.100	0.00
Relatedness Dissatisfaction	Pre	0.70***	0.03	< 0.001	0.91
	SL vs. No SL	-0.03	0.06	0.625	0.00
Autonomy Satisfaction	Pre	0.69***	0.03	< 0.001	0.81
	SL vs. No SL	-0.01	0.06	0.842	0.00
Autonomy Dissatisfaction	Pre	0.73***	0.03	< 0.001	0.98
	SL vs. No SL	0.05	0.06	0.446	0.00
Competency Satisfaction	Pre	0.70***	0.03	< 0.001	0.90
	SL vs. No SL	0.02	0.06	0.782	0.00
Competency Dissatisfaction	Pre	0.68***	0.03	< 0.001	0.95
	SL vs. No SL	-0.04	0.06	0.544	0.00
Relatedness Overall	Pre	0.83***	0.03	< 0.001	1.50
	SL vs. No SL	0.09	0.06	0.120	0.00
Autonomy Overall	Pre	0.80***	0.03	< 0.001	1.33
	SL vs. No SL	-0.03	0.06	0.625	0.00
Competency Overall	Pre	0.78***	0.03	< 0.001	1.51
	SL vs. No SL	0.04	0.05	0.427	0.00

Note. Estimates represent unstandardized regression weights in pre-intervention standard deviation units. Effect size is Cohen's f^2 and can be interpreted as small = 0.02, medium = 0.15, large = 0.35.

Supplementary Table S7.

Slow Looking (1) vs. no slow looking (0) and the condition and pre-test flourishing interaction predicting post-test flourishing.

**Variable**	**Predictor**	**Estimate**	***SE***	***p***	**Effect Size**
Support	Pre	0.80***	0.03	< 0.001	1.25
	SL	0.04	0.05	0.39	0.00
	Interaction	-0.02	0.05	0.628	0.00
Community	Pre	0.77***	0.03	< 0.001	1.00
	SL	-0.03	0.05	0.61	0.00
	Interaction	0.04	0.05	0.449	0.00
Trust	Pre	0.83***	0.03	< 0.001	1.07
	SL	0.05	0.05	0.322	0.00
	Interaction	-0.04	0.05	0.38	0.00
Respect	Pre	0.81***	0.03	< 0.001	1.16
	SL	0.02	0.05	0.71	0.00
	Interaction	-0.06	0.05	0.231	0.00
Loneliness	Pre	0.86***	0.03	< 0.001	1.59
	SL	0.00	0.04	0.957	0.00
	Interaction	0.02	0.04	0.704	0.00
Belonging	Pre	0.83***	0.03	< 0.001	1.19
	SL	0.02	0.05	0.728	0.00
	Interaction	-0.01	0.05	0.912	0.00
Engagement	Pre	0.69***	0.04	< 0.001	0.53
	SL	0.04	0.06	0.505	0.00
	Interaction	-0.04	0.06	0.554	0.00
Skills	Pre	0.75***	0.03	< 0.001	0.85
	SL	-0.09	0.05	0.078	0.00
	Interaction	0.01	0.05	0.829	0.00
Learning	Pre	0.61***	0.04	< 0.001	0.41
	SL	-0.03	0.06	0.581	0.00
	Interaction	0.01	0.06	0.812	0.00
Accomplishment	Pre	0.88***	0.02	< 0.001	1.85
	SL	0.09*	0.04	0.024	0.01
	Interaction	0.01	0.04	0.782	0.01
Self-Efficacy	Pre	0.83***	0.03	< 0.001	1.19
	SL	-0.02	0.05	0.669	0.00
	Interaction	0.04	0.05	0.377	0.00
Self-Worth	Pre	0.78***	0.03	< 0.001	1.05
	SL	-0.04	0.05	0.419	0.00
	Interaction	0.05	0.05	0.349	0.00
Control	Pre	0.70***	0.04	< 0.001	0.55
	SL	0.08	0.06	0.212	0.00

	Interaction	0.03	0.07	0.64	0.00
Meaning	Pre	0.86***	0.02	< 0.001	1.76
	SL	-0.03	0.04	0.517	0.00
	Interaction	0.01	0.04	0.87	0.00
Optimism	Pre	0.83***	0.02	< 0.001	1.74
	SL	0.01	0.04	0.812	0.00
	Interaction	0.04	0.04	0.326	0.00
Life Satisfaction	Pre	0.89***	0.02	< 0.001	2.25
	SL	0.00	0.04	0.998	0.00
	Interaction	-0.01	0.04	0.812	0.00
Positive Feelings	Pre	0.85***	0.03	< 0.001	1.65
	SL	0.01	0.04	0.81	0.00
	Interaction	0.04	0.04	0.298	0.00
Negative Feelings	Pre	0.81***	0.03	< 0.001	1.33
	SL	0.00	0.04	0.956	0.00
	Interaction	0.11*	0.04	0.016	0.00
Overall Thriving	Pre	0.85***	0.02	< 0.001	1.73
	SL	0.02	0.04	0.601	0.00
	Interaction	0.07	0.04	0.109	0.00
Stress	Pre	0.81***	0.03	< 0.001	1.21
	SL	0.03	0.05	0.488	0.00
	Interaction	0.03	0.05	0.564	0.00
Depression	Pre	0.85***	0.03	< 0.001	1.58
	SL	0.02	0.04	0.714	0.00
	Interaction	0.00	0.04	0.938	0.00
Anxiety	Pre	0.86***	0.03	< 0.001	1.37
	SL	0.01	0.05	0.809	0.00
	Interaction	-0.01	0.05	0.861	0.00
Relatedness Satisfaction	Pre	0.78***	0.03	< 0.001	0.80
	SL	0.06	0.06	0.252	0.00
	Interaction	0.00	0.06	1	0.00
Relatedness Dissatisfaction	Pre	0.71***	0.03	< 0.001	0.63
	SL	-0.04	0.06	0.518	0.00
	Interaction	0.00	0.06	0.967	0.00
Autonomy Satisfaction	Pre	0.66***	0.04	< 0.001	0.48
	SL	-0.01	0.06	0.929	0.00
	Interaction	0.06	0.06	0.349	0.00
Autonomy Dissatisfaction	Pre	0.68***	0.03	< 0.001	0.58
	SL	0.03	0.06	0.597	0.00
	Interaction	0.11	0.06	0.063	0.00
Competency Satisfaction	Pre	0.66***	0.03	< 0.001	0.54
	SL	0.00	0.06	0.962	0.00
	Interaction	0.10	0.06	0.108	0.00

Competency Dissatisfaction	Pre	0.66***	0.03	< 0.001	0.60
	SL	-0.02	0.06	0.778	0.00
	Interaction	0.04	0.06	0.461	0.00
Relatedness Overall	Pre	0.83***	0.03	< 0.001	1.00
	SL	0.07	0.05	0.208	0.00
	Interaction	-0.02	0.05	0.705	0.00
Autonomy Overall	Pre	0.76***	0.03	< 0.001	0.78
	SL	-0.02	0.06	0.721	0.00
	Interaction	0.08	0.06	0.166	0.00
Competency Overall	Pre	0.76***	0.03	< 0.001	0.94
	SL	0.02	0.05	0.685	0.00
	Interaction	0.06	0.05	0.255	0.00

Note. Estimates represent unstandardized regression weights in pre-intervention standard deviation units. Effect size is Cohen's f^2 and can be interpreted as small = 0.02, medium = 0.15, large = 0.35.

Supplementary Table S8.

Immersive mindset framing (1) vs. No framing (0) predicting well-being

**Variable**	**Predictor**	***Estimate***	***SE***	***p***	***f***^{2}
Support	Pre	0.79***	0.02	< 0.001	1.79
	Framing vs. No Framing	-0.05	0.05	0.383	0.00
Community	Pre	0.79***	0.03	< 0.001	1.62
	Framing vs. No Framing	0.02	0.06	0.695	0.00
Trust	Pre	0.81***	0.03	< 0.001	1.65
	Framing vs. No Framing	0.04	0.06	0.55	0.00
Respect	Pre	0.77***	0.03	< 0.001	1.59
	Framing vs. No Framing	-0.06	0.06	0.266	0.00
Loneliness	Pre	0.85***	0.02	< 0.001	2.37
	Framing vs. No Framing	0.03	0.05	0.593	0.00
Belonging	Pre	0.84***	0.03	< 0.001	1.85
	Framing vs. No Framing	0.13*	0.06	0.026	0.01
Engagement	Pre	0.66***	0.03	< 0.001	0.82
	Framing vs. No Framing	-0.05	0.07	0.472	0.00
Skills	Pre	0.76***	0.03	< 0.001	1.42
	Framing vs. No Framing	0.12*	0.06	0.047	0.01
Learning	Pre	0.60***	0.03	< 0.001	0.59
	Framing vs. No Framing	-0.05	0.07	0.454	0.00
Accomplishment	Pre	0.87***	0.02	< 0.001	2.85
	Framing vs. No Framing	0.04	0.05	0.468	0.00
Self-Efficacy	Pre	0.84***	0.02	< 0.001	1.94
	Framing vs. No Framing	-0.06	0.06	0.327	0.00
Self-Worth	Pre	0.77***	0.03	< 0.001	1.50
	Framing vs. No Framing	-0.01	0.06	0.915	0.00
Control	Pre	0.68***	0.03	< 0.001	0.77
	Framing vs. No Framing	-0.02	0.07	0.818	0.00
Meaning	Pre	0.87***	0.02	< 0.001	2.78
	Framing vs. No Framing	0.06	0.05	0.198	0.00
Optimism	Pre	0.84***	0.02	< 0.001	2.80
	Framing vs. No Framing	0.04	0.05	0.434	0.00
Life Satisfaction	Pre	0.88***	0.02	< 0.001	3.70
	Framing vs. No Framing	0.08	0.04	0.063	0.01
Positive Feelings	Pre	0.86***	0.02	< 0.001	2.74
	Framing vs. No Framing	0.06	0.05	0.199	0.00
Negative Feelings	Pre	0.84***	0.02	< 0.001	2.23
	Framing vs. No Framing	-0.06	0.05	0.284	0.00
Overall Thriving	Pre	0.86***	0.02	< 0.001	2.76
	Framing vs. No Framing	0.06	0.05	0.223	0.00
Stress	Pre	0.81***	0.03	< 0.001	1.77

	Framing vs. No Framing	0.05	0.06	0.375	0.00
Depression	Pre	0.83***	0.02	< 0.001	2.46
	Framing vs. No Framing	-0.03	0.05	0.525	0.00
Anxiety	Pre	0.83***	0.03	< 0.001	1.77
	Framing vs. No Framing	0.00	0.06	0.947	0.00
Relatedness Satisfaction	Pre	0.77***	0.03	< 0.001	1.22
	Framing vs. No Framing	-0.01	0.07	0.901	0.00
Relatedness Dissatisfaction	Pre	0.70***	0.03	< 0.001	0.91
	Framing vs. No Framing	0.00	0.07	0.998	0.00
Autonomy Satisfaction	Pre	0.69***	0.03	< 0.001	0.81
	Framing vs. No Framing	-0.07	0.07	0.31	0.00
Autonomy Dissatisfaction	Pre	0.73***	0.03	< 0.001	0.99
	Framing vs. No Framing	0.15*	0.07	0.025	0.01
Competency Satisfaction	Pre	0.70***	0.03	< 0.001	0.90
	Framing vs. No Framing	-0.03	0.07	0.712	0.00
Competency Dissatisfaction	Pre	0.68***	0.03	< 0.001	0.95
	Framing vs. No Framing	0.01	0.07	0.858	0.00
Relatedness Overall	Pre	0.82***	0.03	< 0.001	1.49
	Framing vs. No Framing	0.00	0.06	0.994	0.00
Autonomy Overall	Pre	0.80***	0.03	< 0.001	1.34
	Framing vs. No Framing	-0.13*	0.06	0.046	0.01
Competency Overall	Pre	0.78***	0.03	< 0.001	1.50
	Framing vs. No Framing	-0.01	0.06	0.802	0.00

Note. Estimates represent unstandardized regression weights in pre-intervention standard deviation units. Effect size is Cohen's f^2 and can be interpreted as small = 0.02, medium = 0.15, large = 0.35.

Supplementary Table S9.

Immersive mindset framing (1) vs no framing (0) and interaction between condition and pre-test flourishing predicting post-test flourishing.

**Variable**	**Predictor**	**Estimate**	***SE***	***p***	**Effect Size**
Support	Pre	0.80***	0.05	< 0.001	0.43
	Framing	-0.05	0.05	0.392	0.00
	Interaction	-0.01	0.06	0.867	0.00
Community	Pre	0.79***	0.05	< 0.001	0.42
	Framing	0.02	0.06	0.696	0.00
	Interaction	0.00	0.06	0.989	0.00
Trust	Pre	0.81***	0.05	< 0.001	0.48
	Framing	0.04	0.06	0.552	0.00
	Interaction	0.01	0.06	0.894	0.00
Respect	Pre	0.78***	0.05	< 0.001	0.43
	Framing	-0.06	0.06	0.273	0.00
	Interaction	-0.01	0.06	0.857	0.00
Loneliness	Pre	0.76***	0.04	< 0.001	0.54
	Framing	0.02	0.05	0.63	0.00
	Interaction	0.12*	0.05	0.018	0.00
Belonging	Pre	0.92***	0.05	< 0.001	0.54
	Framing	0.13*	0.06	0.023	0.01
	Interaction	-0.11	0.06	0.055	0.01
Engagement	Pre	0.65***	0.06	< 0.001	0.23
	Framing	-0.05	0.07	0.477	0.00
	Interaction	0.03	0.07	0.693	0.00
Skills	Pre	0.73***	0.05	< 0.001	0.36
	Framing	0.12*	0.06	0.049	0.01
	Interaction	0.04	0.06	0.49	0.01
Learning	Pre	0.50***	0.06	< 0.001	0.12
	Framing	-0.06	0.07	0.429	0.00
	Interaction	0.14*	0.07	0.04	0.00
Accomplishment	Pre	0.91***	0.04	< 0.001	0.73
	Framing	0.04	0.05	0.456	0.00
	Interaction	-0.06	0.05	0.27	0.00
Self-Efficacy	Pre	0.91***	0.05	< 0.001	0.64
	Framing	-0.05	0.06	0.357	0.00
	Interaction	-0.10	0.06	0.083	0.00
Self-Worth	Pre	0.72***	0.05	< 0.001	0.34
	Framing	0.00	0.06	0.94	0.00
	Interaction	0.08	0.06	0.19	0.00
Control	Pre	0.77***	0.06	< 0.001	0.25
	Framing	0.00	0.07	0.962	0.00

	Interaction	-0.12	0.07	0.103	0.00
Meaning	Pre	0.85***	0.04	< 0.001	0.75
	Framing	0.06	0.05	0.202	0.00
	Interaction	0.02	0.05	0.671	0.00
Optimism	Pre	0.87***	0.04	< 0.001	0.83
	Framing	0.04	0.05	0.406	0.00
	Interaction	-0.04	0.05	0.357	0.00
Life Satisfaction	Pre	0.90***	0.04	< 0.001	0.96
	Framing	0.08	0.04	0.062	0.01
	Interaction	-0.02	0.04	0.582	0.01
Positive Feelings	Pre	0.88***	0.04	< 0.001	0.75
	Framing	0.06	0.05	0.195	0.00
	Interaction	-0.02	0.05	0.701	0.00
Negative Feelings	Pre	0.81***	0.05	< 0.001	0.52
	Framing	-0.05	0.05	0.304	0.00
	Interaction	0.04	0.05	0.471	0.00
Overall Thriving	Pre	0.87***	0.04	< 0.001	0.69
	Framing	0.06	0.05	0.217	0.00
	Interaction	-0.02	0.05	0.738	0.00
Stress	Pre	0.77***	0.05	< 0.001	0.36
	Framing	0.05	0.06	0.346	0.00
	Interaction	0.05	0.06	0.4	0.00
Depression	Pre	0.77***	0.04	< 0.001	0.53
	Framing	-0.03	0.05	0.594	0.00
	Interaction	0.08	0.05	0.106	0.00
Anxiety	Pre	0.78***	0.06	< 0.001	0.29
	Framing	0.01	0.06	0.884	0.00
	Interaction	0.07	0.07	0.274	0.00
Relatedness Satisfaction	Pre	0.78***	0.06	< 0.001	0.33
	Framing	-0.01	0.07	0.902	0.00
	Interaction	-0.01	0.07	0.865	0.00
Relatedness Dissatisfaction	Pre	0.73***	0.06	< 0.001	0.24
	Framing	0.00	0.07	0.978	0.00
	Interaction	-0.04	0.07	0.552	0.00
Autonomy Satisfaction	Pre	0.64***	0.06	< 0.001	0.19
	Framing	-0.08	0.07	0.288	0.00
	Interaction	0.07	0.07	0.349	0.00
Autonomy Dissatisfaction	Pre	0.72***	0.06	< 0.001	0.22
	Framing	0.15*	0.07	0.025	0.01
	Interaction	0.02	0.07	0.803	0.01
Competency Satisfaction	Pre	0.74***	0.06	< 0.001	0.28
	Framing	-0.02	0.07	0.723	0.00
	Interaction	-0.05	0.07	0.467	0.00

Competency Dissatisfaction	Pre	0.63***	0.06	< 0.001	0.21
	Framing	0.01	0.07	0.826	0.00
	Interaction	0.07	0.07	0.301	0.00
Relatedness Overall	Pre	0.83***	0.06	< 0.001	0.38
	Framing	0.00	0.06	0.998	0.00
	Interaction	-0.01	0.06	0.897	0.00
Autonomy Overall	Pre	0.81***	0.06	< 0.001	0.31
	Framing	-0.13*	0.06	0.046	0.01
	Interaction	0.00	0.07	0.972	0.01
Competency Overall	Pre	0.75***	0.05	< 0.001	0.39
	Framing	-0.02	0.06	0.786	0.00
	Interaction	0.04	0.06	0.531	0.00

Note. Estimates represent unstandardized regression weights in pre-intervention standard deviation units. Effect size is Cohen's f^2 and can be interpreted as small = 0.02, medium = 0.15, large = 0.35.

Supplementary Table S10.

Slow looking, Immersive Mindset Framing, and Interaction predicting well-being

**Variable**	**Predictor**	***Estimate***	***SE***	***p***	***f²***
Support	Pre	0.80***	0.02	< 0.001	1.79
	SL vs. No SL	-0.04	0.10	0.679	0.00
	Framing vs. No Framing	-0.09	0.07	0.229	0.00
	Interaction	0.09	0.11	0.397	0.00
Community	Pre	0.79***	0.03	< 0.001	1.62
	SL vs. No SL	-0.04	0.10	0.730	0.00
	Framing vs. No Framing	0.03	0.08	0.692	0.00
	Interaction	-0.02	0.12	0.866	0.00
Trust	Pre	0.81***	0.03	< 0.001	1.65
	SL vs. No SL	0.15	0.10	0.162	0.00
	Framing vs. No Framing	0.09	0.08	0.236	0.00
	Interaction	-0.14	0.12	0.263	0.00
Respect	Pre	0.77***	0.03	< 0.001	1.59
	SL vs. No SL	0.00	0.10	0.982	0.00
	Framing vs. No Framing	-0.06	0.07	0.391	0.00
	Interaction	0.00	0.12	0.995	0.00
Loneliness	Pre	0.85***	0.02	< 0.001	2.36
	SL vs. No SL	0.01	0.09	0.884	0.00
	Framing vs. No Framing	0.02	0.07	0.766	0.00
	Interaction	0.02	0.11	0.851	0.00
Belonging	Pre	0.84***	0.03	< 0.001	1.85
	SL vs. No SL	0.11	0.10	0.260	0.00
	Framing vs. No Framing	0.19*	0.08	0.013	0.01
	Interaction	-0.14	0.12	0.226	0.00
Engagement	Pre	0.66***	0.03	< 0.001	0.82
	SL vs. No SL	0.09	0.12	0.458	0.00
	Framing vs. No Framing	0.00	0.09	0.976	0.00
	Interaction	-0.11	0.14	0.416	0.00
Skills	Pre	0.76***	0.03	< 0.001	1.43
	SL vs. No SL	-0.17	0.11	0.100	0.00
	Framing vs. No Framing	0.07	0.08	0.386	0.00
	Interaction	0.12	0.12	0.313	0.00
Learning	Pre	0.60***	0.03	< 0.001	0.59
	SL vs. No SL	-0.13	0.13	0.326	0.00
	Framing vs. No Framing	-0.10	0.10	0.278	0.00
	Interaction	0.12	0.15	0.432	0.00
Accomplishment	Pre	0.87***	0.02	< 0.001	2.89
	SL vs. No SL	0.17*	0.09	0.047	0.01
	Framing vs. No Framing	0.08	0.06	0.232	0.00

	Interaction	-0.09	0.10	0.344	0.00
Self-Efficacy	Pre	0.84***	0.03	< 0.001	1.94
	SL vs. No SL	-0.06	0.10	0.521	0.00
	Framing vs. No Framing	-0.08	0.07	0.293	0.00
	Interaction	0.05	0.12	0.648	0.00
Self-Worth	Pre	0.77***	0.03	< 0.001	1.50
	SL vs. No SL	-0.09	0.10	0.383	0.00
	Framing vs. No Framing	-0.02	0.08	0.829	0.00
	Interaction	0.02	0.12	0.855	0.00
Control	Pre	0.69***	0.03	< 0.001	0.78
	SL vs. No SL	0.22	0.13	0.086	0.01
	Framing vs. No Framing	0.08	0.09	0.413	0.00
	Interaction	-0.23	0.15	0.123	0.00
Meaning	Pre	0.87***	0.02	< 0.001	2.78
	SL vs. No SL	-0.10	0.09	0.224	0.00
	Framing vs. No Framing	0.04	0.06	0.572	0.00
	Interaction	0.06	0.10	0.524	0.00
Optimism	Pre	0.84***	0.02	< 0.001	2.81
	SL vs. No SL	-0.07	0.08	0.380	0.00
	Framing vs. No Framing	-0.01	0.06	0.911	0.00
	Interaction	0.11	0.10	0.264	0.00
Life Satisfaction	Pre	0.88***	0.02	< 0.001	3.70
	SL vs. No SL	-0.03	0.08	0.661	0.00
	Framing vs. No Framing	0.05	0.06	0.370	0.00
	Interaction	0.07	0.09	0.400	0.00
Positive Feelings	Pre	0.86***	0.02	< 0.001	2.72
	SL vs. No SL	-0.03	0.09	0.707	0.00
	Framing vs. No Framing	0.04	0.06	0.489	0.00
	Interaction	0.05	0.10	0.643	0.00
Negative Feelings	Pre	0.84***	0.02	< 0.001	2.24
	SL vs. No SL	0.09	0.09	0.326	0.00
	Framing vs. No Framing	-0.01	0.07	0.935	0.00
	Interaction	-0.13	0.11	0.244	0.00
Overall Thriving	Pre	0.86***	0.02	< 0.001	2.75
	SL vs. No SL	-0.01	0.09	0.878	0.00
	Framing vs. No Framing	0.04	0.06	0.499	0.00
	Interaction	0.04	0.10	0.677	0.00
Stress	Pre	0.81***	0.03	< 0.001	1.76
	SL vs. No SL	-0.02	0.10	0.831	0.00
	Framing vs. No Framing	0.02	0.07	0.766	0.00
	Interaction	0.07	0.12	0.540	0.00
Depression	Pre	0.83***	0.02	< 0.001	2.45
	SL vs. No SL	-0.03	0.09	0.750	0.00

	Framing vs. No Framing	-0.06	0.06	0.346	0.00
	Interaction	0.07	0.10	0.468	0.00
Anxiety	Pre	0.83***	0.03	< 0.001	1.77
	SL vs. No SL	-0.08	0.10	0.444	0.00
	Framing vs. No Framing	-0.04	0.08	0.585	0.00
	Interaction	0.11	0.12	0.350	0.00
Relatedness Satisfaction	Pre	0.77***	0.03	< 0.001	1.23
	SL vs. No SL	0.02	0.11	0.884	0.00
	Framing vs. No Framing	-0.05	0.09	0.552	0.00
	Interaction	0.11	0.13	0.417	0.00
Relatedness Dissatisfaction	Pre	0.70***	0.03	< 0.001	0.91
	SL vs. No SL	0.14	0.12	0.237	0.00
	Framing vs. No Framing	0.09	0.09	0.288	0.00
	Interaction	-0.23	0.14	0.095	0.00
Autonomy Satisfaction	Pre	0.69***	0.03	< 0.001	0.80
	SL vs. No SL	0.05	0.13	0.676	0.00
	Framing vs. No Framing	-0.04	0.09	0.697	0.00
	Interaction	-0.09	0.15	0.538	0.00
Autonomy Dissatisfaction	Pre	0.73***	0.03	< 0.001	0.98
	SL vs. No SL	0.02	0.12	0.842	0.00
	Framing vs. No Framing	0.14	0.09	0.116	0.00
	Interaction	0.03	0.14	0.805	0.00
Competency Satisfaction	Pre	0.70***	0.03	< 0.001	0.90
	SL vs. No SL	-0.15	0.12	0.216	0.00
	Framing vs. No Framing	-0.12	0.09	0.192	0.00
	Interaction	0.23	0.14	0.109	0.00
Competency Dissatisfaction	Pre	0.68***	0.03	< 0.001	0.95
	SL vs. No SL	0.04	0.12	0.754	0.00
	Framing vs. No Framing	0.05	0.09	0.554	0.00
	Interaction	-0.10	0.13	0.469	0.00
Relatedness Overall	Pre	0.83***	0.03	< 0.001	1.51
	SL vs. No SL	-0.06	0.11	0.585	0.00
	Framing vs. No Framing	-0.08	0.08	0.324	0.00
	Interaction	0.20	0.13	0.118	0.00
Autonomy Overall	Pre	0.80***	0.03	< 0.001	1.34
	SL vs. No SL	0.01	0.11	0.923	0.00
	Framing vs. No Framing	-0.11	0.08	0.204	0.00
	Interaction	-0.06	0.13	0.670	0.00
Competency Overall	Pre	0.78***	0.03	< 0.001	1.51
	SL vs. No SL	-0.10	0.10	0.319	0.00
	Framing vs. No Framing	-0.10	0.08	0.221	0.00
	Interaction	0.20	0.12	0.103	0.00

Note. Estimates represent unstandardized regression weights in pre-intervention standard deviation units. Effect size is Cohen's f^2 and can be interpreted as small = 0.02, medium = 0.15, large = 0.35.

Supplementary Table S11.

All condition factors and interactions predicting post-test flourishing

**Variable**	**Predictor**	**Estimate**	***SE***	***p***	**Effect Size**
Support	Pre	0.85***	0.07	< 0.001	0.28
	SL vs. No SL	-0.03	0.10	0.773	0.00
	Framing vs. No Framing	-0.08	0.07	0.278	0.00
	Pre * SL	-0.12	0.10	0.228	0.00
	Pre * Frame	-0.06	0.08	0.422	0.00
	SL * Frame	0.08	0.11	0.462	0.00
	Three Way Interaction	0.13	0.12	0.287	0.00
Community	Pre	0.83***	0.07	< 0.001	0.27
	SL vs. No SL	-0.04	0.10	0.7	0.00
	Framing vs. No Framing	0.03	0.08	0.699	0.00
	Pre * SL	-0.08	0.10	0.418	0.00
	Pre * Frame	-0.06	0.08	0.42	0.00
	SL * Frame	-0.02	0.12	0.89	0.00
	Three Way Interaction	0.15	0.12	0.216	0.00
Trust	Pre	0.83***	0.06	< 0.001	0.31
	SL vs. No SL	0.15	0.10	0.162	0.00
	Framing vs. No Framing	0.09	0.08	0.244	0.00
	Pre * SL	-0.07	0.10	0.488	0.00
	Pre * Frame	-0.01	0.08	0.926	0.00
	SL * Frame	-0.13	0.12	0.266	0.00
	Three Way Interaction	0.04	0.12	0.737	0.00
Respect	Pre	0.77***	0.06	< 0.001	0.30
	SL vs. No SL	0.00	0.10	0.966	0.00
	Framing vs. No Framing	-0.06	0.07	0.385	0.00
	Pre * SL	0.05	0.11	0.679	0.00
	Pre * Frame	0.03	0.07	0.704	0.00
	SL * Frame	0.01	0.12	0.964	0.00
	Three Way Interaction	-0.10	0.12	0.415	0.00
Loneliness	Pre	0.71***	0.05	< 0.001	0.29
	SL vs. No SL	0.01	0.09	0.919	0.00
	Framing vs. No Framing	0.02	0.07	0.792	0.00
	Pre * SL	0.14	0.09	0.121	0.00
	Pre * Frame	0.16*	0.06	0.012	0.01
	SL * Frame	0.02	0.10	0.857	0.00
	Three Way Interaction	-0.12	0.10	0.246	0.00
Belonging	Pre	0.92***	0.07	< 0.001	0.32
	SL vs. No SL	0.11	0.10	0.262	0.00
	Framing vs. No Framing	0.19*	0.08	0.012	0.01
	Pre * SL	0.01	0.11	0.933	0.00

	Pre * Frame	-0.11	0.08	0.174	0.00
	SL * Frame	-0.15	0.12	0.22	0.00
	Three Way Interaction	-0.02	0.12	0.841	0.00
Engagement	Pre	0.73***	0.08	< 0.001	0.14
	SL vs. No SL	0.08	0.12	0.496	0.00
	Framing vs. No Framing	0.00	0.09	0.967	0.00
	Pre * SL	-0.15	0.11	0.176	0.00
	Pre * Frame	-0.07	0.09	0.461	0.00
	SL * Frame	-0.11	0.14	0.447	0.00
	Three Way Interaction	0.19	0.13	0.163	0.00
Skills	Pre	0.72***	0.07	< 0.001	0.18
	SL vs. No SL	-0.17	0.11	0.1	0.00
	Framing vs. No Framing	0.07	0.08	0.398	0.00
	Pre * SL	0.03	0.10	0.781	0.00
	Pre * Frame	0.05	0.08	0.512	0.00
	SL * Frame	0.13	0.12	0.309	0.00
	Three Way Interaction	-0.02	0.12	0.836	0.00
Learning	Pre	0.45***	0.08	< 0.001	0.05
	SL vs. No SL	-0.13	0.13	0.3	0.00
	Framing vs. No Framing	-0.11	0.09	0.247	0.00
	Pre * SL	0.1	0.12	0.409	0.00
	Pre * Frame	0.18	0.10	0.06	0.01
	SL * Frame	0.12	0.15	0.41	0.00
	Three Way Interaction	-0.06	0.14	0.667	0.00
Accomplishment	Pre	0.90***	0.06	< 0.001	0.44
	SL vs. No SL	0.17*	0.09	0.044	0.01
	Framing vs. No Framing	0.08	0.06	0.218	0.00
	Pre * SL	0.04	0.09	0.623	0.00
	Pre * Frame	-0.05	0.06	0.424	0.00
	SL * Frame	-0.10	0.10	0.328	0.00
	Three Way Interaction	-0.01	0.10	0.891	0.00
Self-Efficacy	Pre	0.83***	0.07	< 0.001	0.28
	SL vs. No SL	-0.07	0.10	0.483	0.00
	Framing vs. No Framing	-0.08	0.07	0.294	0.00
	Pre * SL	0.16	0.09	0.096	0.00
	Pre * Frame	-0.02	0.08	0.775	0.00
	SL * Frame	0.06	0.12	0.622	0.00
	Three Way Interaction	-0.16	0.11	0.166	0.00
Self-Worth	Pre	0.67***	0.07	< 0.001	0.18
	SL vs. No SL	-0.09	0.10	0.408	0.00
	Framing vs. No Framing	-0.01	0.08	0.879	0.00
	Pre * SL	0.12	0.10	0.257	0.00
	Pre * Frame	0.09	0.08	0.231	0.00

	SL * Frame	0.02	0.12	0.888	0.00
	Three Way Interaction	-0.03	0.12	0.811	0.00
Control	Pre	0.78***	0.08	< 0.001	0.15
	SL vs. No SL	0.22	0.13	0.081	0.01
	Framing vs. No Framing	0.09	0.10	0.333	0.00
	Pre * SL	-0.01	0.13	0.962	0.00
	Pre * Frame	-0.15	0.09	0.112	0.00
	SL * Frame	-0.23	0.15	0.12	0.00
	Three Way Interaction	0.08	0.15	0.604	0.00
Meaning	Pre	0.85***	0.05	< 0.001	0.44
	SL vs. No SL	-0.10	0.09	0.233	0.00
	Framing vs. No Framing	0.04	0.06	0.568	0.00
	Pre * SL	0.01	0.08	0.928	0.00
	Pre * Frame	0.02	0.06	0.762	0.00
	SL * Frame	0.06	0.10	0.535	0.00
	Three Way Interaction	-0.01	0.10	0.948	0.00
Optimism	Pre	0.86***	0.05	< 0.001	0.45
	SL vs. No SL	-0.07	0.08	0.373	0.00
	Framing vs. No Framing	0.00	0.06	0.937	0.00
	Pre * SL	0.03	0.08	0.71	0.00
	Pre * Frame	-0.05	0.06	0.394	0.00
	SL * Frame	0.11	0.10	0.259	0.00
	Three Way Interaction	0.03	0.09	0.772	0.00
Life Satisfaction	Pre	0.90***	0.05	< 0.001	0.57
	SL vs. No SL	-0.03	0.08	0.677	0.00
	Framing vs. No Framing	0.05	0.06	0.36	0.00
	Pre * SL	-0.01	0.08	0.925	0.00
	Pre * Frame	-0.02	0.06	0.72	0.00
	SL * Frame	0.07	0.09	0.417	0.00
	Three Way Interaction	0.00	0.09	0.976	0.00
Positive Feelings	Pre	0.85***	0.05	< 0.001	0.42
	SL vs. No SL	-0.03	0.09	0.692	0.00
	Framing vs. No Framing	0.05	0.06	0.476	0.00
	Pre * SL	0.06	0.09	0.465	0.00
	Pre * Frame	-0.02	0.06	0.78	0.00
	SL * Frame	0.05	0.10	0.627	0.00
	Three Way Interaction	-0.01	0.10	0.955	0.00
Negative Feelings	Pre	0.75***	0.06	< 0.001	0.26
	SL vs. No SL	0.10	0.09	0.26	0.00
	Framing vs. No Framing	0.00	0.07	0.988	0.00
	Pre * SL	0.15	0.09	0.108	0.00
	Pre * Frame	0.05	0.07	0.48	0.00
	SL * Frame	-0.15	0.11	0.172	0.00

	Three Way Interaction	-0.03	0.11	0.804	0.00
Overall Thriving	Pre	0.83***	0.06	< 0.001	0.36
	SL vs. No SL	-0.02	0.09	0.827	0.00
	Framing vs. No Framing	0.04	0.06	0.517	0.00
	Pre * SL	0.10	0.09	0.269	0.00
	Pre * Frame	-0.01	0.07	0.865	0.00
	SL * Frame	0.05	0.10	0.612	0.00
	Three Way Interaction	0.00	0.10	0.963	0.00
Stress	Pre	0.83***	0.07	< 0.001	0.24
	SL vs. No SL	-0.03	0.10	0.738	0.00
	Framing vs. No Framing	0.02	0.07	0.783	0.00
	Pre * SL	-0.13	0.11	0.236	0.00
	Pre * Frame	-0.04	0.08	0.641	0.00
	SL * Frame	0.08	0.12	0.492	0.00
	Three Way Interaction	0.21	0.12	0.08	0.01
Depression	Pre	0.80***	0.06	< 0.001	0.32
	SL vs. No SL	-0.03	0.09	0.742	0.00
	Framing vs. No Framing	-0.06	0.07	0.37	0.00
	Pre * SL	-0.07	0.09	0.407	0.00
	Pre * Frame	0.01	0.07	0.873	0.00
	SL * Frame	0.07	0.10	0.498	0.00
	Three Way Interaction	0.16	0.10	0.125	0.00
Anxiety	Pre	0.75***	0.08	< 0.001	0.15
	SL vs. No SL	-0.08	0.10	0.459	0.00
	Framing vs. No Framing	-0.04	0.08	0.631	0.00
	Pre * SL	0.06	0.12	0.603	0.00
	Pre * Frame	0.09	0.09	0.292	0.00
	SL * Frame	0.11	0.12	0.366	0.00
	Three Way Interaction	-0.04	0.14	0.757	0.00
Relatedness Satisfaction	Pre	0.76***	0.08	< 0.001	0.17
	SL vs. No SL	0.02	0.12	0.876	0.00
	Framing vs. No Framing	-0.05	0.09	0.546	0.00
	Pre * SL	0.04	0.11	0.731	0.00
	Pre * Frame	0.01	0.09	0.866	0.00
	SL * Frame	0.11	0.13	0.426	0.00
	Three Way Interaction	-0.05	0.13	0.708	0.00
Relatedness Dissatisfaction	Pre	0.69***	0.08	< 0.001	0.12
	SL vs. No SL	0.14	0.12	0.236	0.00
	Framing vs. No Framing	0.10	0.09	0.284	0.00
	Pre * SL	0.10	0.13	0.435	0.00
	Pre * Frame	0.01	0.09	0.886	0.00

	SL * Frame	-0.23	0.14	0.098	0.00
	Three Way Interaction	-0.12	0.15	0.414	0.00
Autonomy Satisfaction	Pre	0.55***	0.08	< 0.001	0.08
	SL vs. No SL	0.04	0.13	0.77	0.00
	Framing vs. No Framing	-0.05	0.09	0.601	0.00
	Pre * SL	0.22	0.12	0.078	0.01
	Pre * Frame	0.16	0.09	0.08	0.01
	SL * Frame	-0.07	0.15	0.619	0.00
	Three Way Interaction	-0.24	0.14	0.103	0.00
Autonomy Dissatisfaction	Pre	0.63***	0.08	< 0.001	0.11
	SL vs. No SL	0.04	0.12	0.737	0.00
	Framing vs. No Framing	0.14	0.09	0.116	0.00
	Pre * SL	0.27*	0.14	0.047	0.01
	Pre * Frame	0.09	0.09	0.346	0.00
	SL * Frame	0.02	0.14	0.899	0.00
	Three Way Interaction	-0.21	0.15	0.163	0.00
Competency Satisfaction	Pre	0.55***	0.08	< 0.001	0.09
	SL vs. No SL	-0.16	0.12	0.186	0.00
	Framing vs. No Framing	-0.12	0.09	0.165	0.00
	Pre * SL	0.44***	0.12	< 0.001	0.02
	Pre * Frame	0.16	0.09	0.074	0.01
	SL * Frame	0.23	0.14	0.096	0.00
	Three Way Interaction	-0.49***	0.14	< 0.001	0.02
Competency Dissatisfaction	Pre	0.66***	0.07	< 0.001	0.14
	SL vs. No SL	0.03	0.12	0.783	0.00
	Framing vs. No Framing	0.05	0.09	0.545	0.00
	Pre * SL	-0.08	0.12	0.479	0.00
	Pre * Frame	0.01	0.08	0.866	0.00
	SL * Frame	-0.10	0.13	0.46	0.00
	Three Way Interaction	0.15	0.14	0.263	0.00
Relatedness Overall	Pre	0.83***	0.07	< 0.001	0.22
	SL vs. No SL	-0.06	0.11	0.587	0.00
	Framing vs. No Framing	-0.08	0.08	0.322	0.00
	Pre * SL	0.00	0.11	0.994	0.00
	Pre * Frame	0.01	0.08	0.872	0.00
	SL * Frame	0.20	0.13	0.121	0.00
	Three Way Interaction	-0.03	0.13	0.805	0.00
Autonomy Overall	Pre	0.72***	0.08	< 0.001	0.15
	SL vs. No SL	0.00	0.11	0.973	0.00
	Framing vs. No Framing	-0.11	0.08	0.19	0.00

	Pre * SL	0.20	0.12	0.095	0.00
	Pre * Frame	0.07	0.09	0.412	0.00
	SL * Frame	-0.04	0.13	0.754	0.00
	Three Way Interaction	-0.19	0.14	0.173	0.00
Competency Overall	Pre	0.68***	0.07	< 0.001	0.18
	SL vs. No SL	-0.11	0.10	0.276	0.00
	Framing vs. No Framing	-0.10	0.08	0.188	0.00
	Pre * SL	0.17	0.10	0.087	0.01
	Pre * Frame	0.11	0.08	0.152	0.00
	SL * Frame	0.21	0.12	0.084	0.01
	Three Way Interaction	-0.16	0.12	0.185	0.00

Note. Estimates represent unstandardized regression weights in pre-intervention standard deviation units. Effect size is Cohen's f^2 and can be interpreted as small = 0.02, medium = 0.15, large = 0.35.

Supplementary Table S12.

Latent immersion factor predicting well-being

**Variable**	**Predictor**	**Estimate**	***SE***	***p***	**Effect Size**
Support	Pre	0.80***	0.02	< 0.001	1.76
	Immersion	0.01	0.02	0.652	0.00
	Interaction	0.04	0.02	0.06	0.00
Community	Pre	0.78***	0.03	< 0.001	1.52
	Immersion	0.03	0.02	0.202	0.00
	Interaction	-0.01	0.02	0.741	0.00
Trust	Pre	0.81***	0.03	< 0.001	1.61
	Immersion	-0.01	0.02	0.77	0.00
	Interaction	0.04	0.02	0.105	0.00
Respect	Pre	0.77***	0.03	< 0.001	1.57
	Immersion	0.05*	0.02	0.031	0.01
	Interaction	0.01	0.02	0.768	0.01
Loneliness	Pre	0.84***	0.02	< 0.001	2.32
	Immersion	-0.02	0.02	0.307	0.00
	Interaction	0.01	0.02	0.689	0.00
Belonging	Pre	0.83***	0.03	< 0.001	1.74
	Immersion	0.04	0.02	0.123	0.00
	Interaction	-0.03	0.02	0.217	0.00
Engagement	Pre	0.63***	0.03	< 0.001	0.70
	Immersion	0.13***	0.03	< 0.001	0.03
	Interaction	-0.01	0.03	0.673	0.03
Skills	Pre	0.76***	0.03	< 0.001	1.34
	Immersion	0.02	0.03	0.505	0.00
	Interaction	-0.01	0.02	0.661	0.00
Learning	Pre	0.58***	0.03	< 0.001	0.54
	Immersion	0.11***	0.03	< 0.001	0.02
	Interaction	0.02	0.03	0.458	0.02
Accomplishment	Pre	0.87***	0.02	< 0.001	2.82
	Immersion	0.03	0.02	0.142	0.00
	Interaction	0.00	0.02	0.887	0.00
Self-Efficacy	Pre	0.84***	0.03	< 0.001	1.89
	Immersion	0.01	0.02	0.638	0.00
	Interaction	-0.02	0.03	0.414	0.00
Self-Worth	Pre	0.77***	0.03	< 0.001	1.48
	Immersion	0.01	0.02	0.741	0.00
	Interaction	0.04	0.02	0.063	0.00
Control	Pre	0.68***	0.03	< 0.001	0.77
	Immersion	0.03	0.03	0.355	0.00
	Interaction	0.04	0.03	0.206	0.00

Meaning	Pre	0.86***	0.02	< 0.001	2.72
	Immersion	0.04*	0.02	0.046	0.01
	Interaction	-0.02	0.02	0.326	0.01
Optimism	Pre	0.84***	0.02	< 0.001	2.74
	Immersion	0.01	0.02	0.647	0.00
	Interaction	-0.01	0.02	0.644	0.00
Life Satisfaction	Pre	0.88***	0.02	< 0.001	3.66
	Immersion	0.02	0.02	0.192	0.00
	Interaction	0.02	0.02	0.308	0.00
Positive Feelings	Pre	0.86***	0.02	< 0.001	2.67
	Immersion	0.01	0.02	0.696	0.00
	Interaction	0.00	0.02	0.86	0.00
Negative Feelings	Pre	0.84***	0.02	< 0.001	2.22
	Immersion	-0.03	0.02	0.171	0.00
	Interaction	-0.01	0.02	0.696	0.00
Overall Thriving	Pre	0.86***	0.02	< 0.001	2.69
	Immersion	0.01	0.02	0.567	0.00
	Interaction	0.02	0.02	0.315	0.00
Stress	Pre	0.81***	0.03	< 0.001	1.72
	Immersion	0.05*	0.02	0.048	0.01
	Interaction	0.01	0.03	0.592	0.01
Depression	Pre	0.83***	0.02	< 0.001	2.47
	Immersion	0.02	0.02	0.289	0.00
	Interaction	0.02	0.02	0.293	0.00
Anxiety	Pre	0.82***	0.03	< 0.001	1.64
	Immersion	0.05*	0.02	0.042	0.01
	Interaction	0.04	0.03	0.178	0.01
Relatedness Satisfaction	Pre	0.77***	0.03	< 0.001	1.17
	Immersion	0.00	0.03	0.966	0.00
	Interaction	0.01	0.03	0.831	0.00
Relatedness Dissatisfaction	Pre	0.70***	0.03	< 0.001	0.90
	Immersion	-0.03	0.03	0.339	0.00
	Interaction	0.00	0.03	0.885	0.00
Autonomy Satisfaction	Pre	0.68***	0.03	< 0.001	0.79
	Immersion	0.09**	0.03	0.003	0.02
	Interaction	-0.03	0.03	0.387	0.02
Autonomy Dissatisfaction	Pre	0.73***	0.03	< 0.001	0.96
	Immersion	-0.01	0.03	0.855	0.00
	Interaction	0.03	0.03	0.274	0.00
Competency Satisfaction	Pre	0.70***	0.03	< 0.001	0.86
	Immersion	0.02	0.03	0.521	0.00
	Interaction	0.04	0.03	0.152	0.00
Competency Dissatisfaction	Pre	0.68***	0.03	< 0.001	0.94

	Immersion	0.04	0.03	0.129	0.00
	Interaction	0.05	0.03	0.053	0.00
Relatedness Overall	Pre	0.82***	0.03	< 0.001	1.46
	Immersion	0.01	0.03	0.74	0.00
	Interaction	-0.02	0.03	0.482	0.00
Autonomy Overall	Pre	0.80***	0.03	< 0.001	1.32
	Immersion	0.05	0.03	0.067	0.01
	Interaction	-0.02	0.03	0.502	0.01
Competency Overall	Pre	0.78***	0.03	< 0.001	1.50
	Immersion	-0.03	0.02	0.254	0.00
	Interaction	0.07**	0.03	0.008	0.00

Note. Estimates represent unstandardized regression weights in pre-intervention standard deviation units. Effect size is Cohen's f^2 and can be interpreted as small = 0.02, medium = 0.15, large = 0.35.

Supplementary Table S13.

Latent art topic factor predicting well-being

**Variable**	**Predictor**	**Estimate**	***SE***	***p***	**Effect Size**
Support	Pre	0.80***	0.02	< 0.001	1.79
	Art Topics	-0.02	0.24	0.932	0.00
	Interaction	0.21	0.25	0.402	0.00
Community	Pre	0.80***	0.03	< 0.001	1.62
	Art Topics	-0.56*	0.26	0.028	0.01
	Interaction	0.14	0.26	0.588	0.01
Trust	Pre	0.81***	0.03	< 0.001	1.63
	Art Topics	0.47	0.26	0.073	0.01
	Interaction	-0.18	0.27	0.515	0.01
Respect	Pre	0.77***	0.03	< 0.001	1.58
	Art Topics	0.22	0.25	0.379	0.00
	Interaction	-0.18	0.27	0.506	0.00
Loneliness	Pre	0.85***	0.02	< 0.001	2.38
	Art Topics	-0.27	0.23	0.228	0.00
	Interaction	0.21	0.23	0.375	0.00
Belonging	Pre	0.84***	0.03	< 0.001	1.80
	Art Topics	-0.17	0.26	0.51	0.00
	Interaction	-0.08	0.26	0.742	0.00
Engagement	Pre	0.66***	0.03	< 0.001	0.80
	Art Topics	-0.06	0.30	0.84	0.00
	Interaction	-0.51	0.32	0.111	0.00
Skills	Pre	0.76***	0.03	< 0.001	1.41
	Art Topics	-0.01	0.27	0.956	0.00
	Interaction	-0.12	0.27	0.668	0.00
Learning	Pre	0.61***	0.03	< 0.001	0.59
	Art Topics	0.10	0.32	0.754	0.00
	Interaction	0.46	0.30	0.132	0.00
Accomplishment	Pre	0.87***	0.02	< 0.001	2.85
	Art Topics	0.07	0.22	0.737	0.00
	Interaction	-0.06	0.21	0.778	0.00
Self-Efficacy	Pre	0.84***	0.03	< 0.001	1.93
	Art Topics	0.07	0.25	0.788	0.00
	Interaction	0.06	0.25	0.808	0.00
Self-Worth	Pre	0.77***	0.03	< 0.001	1.49
	Art Topics	-0.07	0.26	0.79	0.00
	Interaction	-0.20	0.26	0.437	0.00
Control	Pre	0.68***	0.03	< 0.001	0.77
	Art Topics	-0.27	0.32	0.394	0.00
	Interaction	-0.13	0.34	0.695	0.00

Meaning	Pre	0.87***	0.02	< 0.001	2.77
	Art Topics	0.19	0.22	0.386	0.00
	Interaction	0.15	0.21	0.48	0.00
Optimism	Pre	0.84***	0.02	< 0.001	2.78
	Art Topics	0.13	0.21	0.52	0.00
	Interaction	-0.18	0.21	0.383	0.00
Life Satisfaction	Pre	0.88***	0.02	< 0.001	3.69
	Art Topics	-0.10	0.19	0.618	0.00
	Interaction	-0.31	0.19	0.105	0.00
Positive Feelings	Pre	0.86***	0.02	< 0.001	2.73
	Art Topics	0.29	0.21	0.172	0.00
	Interaction	-0.41	0.22	0.07	0.00
Negative Feelings	Pre	0.84***	0.02	< 0.001	2.27
	Art Topics	-0.61**	0.23	0.009	0.01
	Interaction	-0.32	0.24	0.18	0.01
Overall Thriving	Pre	0.86***	0.02	< 0.001	2.77
	Art Topics	0.44*	0.21	0.038	0.01
	Interaction	-0.37	0.22	0.093	0.01
Stress	Pre	0.81***	0.03	< 0.001	1.76
	Art Topics	-0.44	0.25	0.083	0.01
	Interaction	-0.49	0.27	0.067	0.01
Depression	Pre	0.83***	0.02	< 0.001	2.45
	Art Topics	-0.23	0.22	0.297	0.00
	Interaction	-0.38	0.23	0.107	0.00
Anxiety	Pre	0.83***	0.03	< 0.001	1.76
	Art Topics	-0.40	0.26	0.117	0.00
	Interaction	-0.57*	0.28	0.044	0.00
Relatedness Satisfaction	Pre	0.77***	0.03	< 0.001	1.22
	Art Topics	0.05	0.29	0.859	0.00
	Interaction	0.02	0.28	0.942	0.00
Relatedness Dissatisfaction	Pre	0.70***	0.03	< 0.001	0.91
	Art Topics	-0.21	0.30	0.475	0.00
	Interaction	-0.47	0.30	0.124	0.00
Autonomy Satisfaction	Pre	0.69***	0.03	< 0.001	0.80
	Art Topics	0.10	0.32	0.745	0.00
	Interaction	-0.02	0.33	0.957	0.00
Autonomy Dissatisfaction	Pre	0.73***	0.03	< 0.001	0.98
	Art Topics	0.16	0.30	0.6	0.00
	Interaction	-0.18	0.30	0.548	0.00
Competency Satisfaction	Pre	0.70***	0.03	< 0.001	0.90
	Art Topics	0.15	0.30	0.613	0.00
	Interaction	0.37	0.29	0.21	0.00
Competency Dissatisfaction	Pre	0.68***	0.03	< 0.001	0.95

	Art Topics	0.03	0.29	0.931	0.00
	Interaction	-0.56	0.29	0.053	0.00
Relatedness Overall	Pre	0.82***	0.03	< 0.001	1.49
	Art Topics	0.13	0.28	0.641	0.00
	Interaction	-0.16	0.28	0.574	0.00
Autonomy Overall	Pre	0.80***	0.03	< 0.001	1.33
	Art Topics	-0.08	0.28	0.771	0.00
	Interaction	0.00	0.29	0.997	0.00
Competency Overall	Pre	0.78***	0.03	< 0.001	1.51
	Art Topics	0.11	0.26	0.682	0.00
	Interaction	-0.34	0.26	0.193	0.00

Note. Estimates represent unstandardized regression weights in pre-intervention standard deviation units. Effect size is Cohen's f^2 and can be interpreted as small = 0.02, medium = 0.15, large = 0.35.

Supplementary Table S14.

Latent feeling topic factor predicting well-being

**Variable**	**Predictor**	**Estimate**	***SE***	***p***	**Effect Size**
Support	Pre	0.79***	0.02	< 0.001	1.79
	Feeling Topics	-0.24	0.23	0.297	0.00
	Interaction	0.15	0.24	0.531	0.00
Community	Pre	0.79***	0.03	< 0.001	1.61
	Feeling Topics	0.13	0.25	0.613	0.00
	Interaction	0.06	0.24	0.813	0.00
Trust	Pre	0.81***	0.03	< 0.001	1.63
	Feeling Topics	-0.23	0.25	0.354	0.00
	Interaction	-0.15	0.25	0.541	0.00
Respect	Pre	0.77***	0.03	< 0.001	1.56
	Feeling Topics	-0.37	0.24	0.125	0.00
	Interaction	0.37	0.25	0.142	0.00
Loneliness	Pre	0.85***	0.02	< 0.001	2.39
	Feeling Topics	0.42	0.22	0.054	0.01
	Interaction	-0.01	0.23	0.957	0.01
Belonging	Pre	0.84***	0.03	< 0.001	1.80
	Feeling Topics	-0.13	0.25	0.607	0.00
	Interaction	0.01	0.24	0.953	0.00
Engagement	Pre	0.66***	0.03	< 0.001	0.80
	Feeling Topics	-0.07	0.29	0.814	0.00
	Interaction	0.05	0.30	0.876	0.00
Skills	Pre	0.76***	0.03	< 0.001	1.41
	Feeling Topics	-0.03	0.26	0.918	0.00
	Interaction	0.3	0.26	0.266	0.00
Learning	Pre	0.60***	0.03	< 0.001	0.59
	Feeling Topics	-0.05	0.31	0.881	0.00
	Interaction	-0.01	0.28	0.985	0.00
Accomplishment	Pre	0.87***	0.02	< 0.001	2.85
	Feeling Topics	-0.21	0.21	0.32	0.00
	Interaction	-0.01	0.21	0.951	0.00
Self-Efficacy	Pre	0.84***	0.03	< 0.001	1.93
	Feeling Topics	-0.21	0.24	0.372	0.00
	Interaction	-0.06	0.24	0.79	0.00
Self-Worth	Pre	0.77***	0.03	< 0.001	1.52
	Feeling Topics	0.05	0.25	0.857	0.00
	Interaction	0.76**	0.25	0.003	0.00
Control	Pre	0.69***	0.03	< 0.001	0.77
	Feeling Topics	0.08	0.31	0.793	0.00
	Interaction	0.02	0.32	0.945	0.00

Meaning	Pre	0.87***	0.02	< 0.001	2.77
	Feeling Topics	-0.28	0.21	0.174	0.00
	Interaction	0.02	0.20	0.939	0.00
Optimism	Pre	0.84***	0.02	< 0.001	2.80
	Feeling Topics	-0.18	0.20	0.379	0.00
	Interaction	0.25	0.20	0.213	0.00
Life Satisfaction	Pre	0.88***	0.02	< 0.001	3.68
	Feeling Topics	-0.11	0.18	0.545	0.00
	Interaction	0.16	0.19	0.406	0.00
Positive Feelings	Pre	0.86***	0.02	< 0.001	2.75
	Feeling Topics	-0.52*	0.21	0.012	0.01
	Interaction	0.13	0.21	0.537	0.01
Negative Feelings	Pre	0.84***	0.02	< 0.001	2.27
	Feeling Topics	0.8***	0.22	< 0.001	0.02
	Interaction	0.37	0.22	0.101	0.02
Overall Thriving	Pre	0.86***	0.02	< 0.001	2.77
	Feeling Topics	-0.65**	0.20	0.002	0.02
	Interaction	0.44*	0.21	0.034	0.02
Stress	Pre	0.81***	0.03	< 0.001	1.73
	Feeling Topics	0.66**	0.24	0.006	0.01
	Interaction	0.40	0.25	0.117	0.01
Depression	Pre	0.82***	0.02	< 0.001	2.41
	Feeling Topics	0.49*	0.21	0.02	0.01
	Interaction	0.53*	0.22	0.015	0.01
Anxiety	Pre	0.83***	0.03	< 0.001	1.69
	Feeling Topics	0.18	0.25	0.462	0.00
	Interaction	0.31	0.26	0.232	0.00
Relatedness Satisfaction	Pre	0.77***	0.03	< 0.001	1.23
	Feeling Topics	-0.51	0.28	0.068	0.01
	Interaction	0.2	0.28	0.466	0.01
Relatedness Dissatisfaction	Pre	0.70***	0.03	< 0.001	0.90
	Feeling Topics	0.35	0.29	0.232	0.00
	Interaction	0.27	0.28	0.339	0.00
Autonomy Satisfaction	Pre	0.68***	0.03	< 0.001	0.78
	Feeling Topics	-0.43	0.30	0.162	0.00
	Interaction	0.38	0.31	0.223	0.00
Autonomy Dissatisfaction	Pre	0.73***	0.03	< 0.001	0.97
	Feeling Topics	0.26	0.29	0.362	0.00
	Interaction	0.31	0.29	0.286	0.00
Competency Satisfaction	Pre	0.70***	0.03	< 0.001	0.90
	Feeling Topics	-0.32	0.29	0.278	0.00
	Interaction	-0.12	0.29	0.688	0.00
Competency Dissatisfaction	Pre	0.68***	0.03	< 0.001	0.94

	Feeling Topics	0.28	0.28	0.314	0.00
	Interaction	0.54	0.28	0.051	0.00
Relatedness Overall	Pre	0.82***	0.03	< 0.001	1.49
	Feeling Topics	-0.46	0.26	0.082	0.01
	Interaction	0.28	0.26	0.284	0.01
Autonomy Overall	Pre	0.80***	0.03	< 0.001	1.30
	Feeling Topics	-0.33	0.27	0.224	0.00
	Interaction	0.19	0.28	0.485	0.00
Competency Overall	Pre	0.78***	0.03	< 0.001	1.50
	Feeling Topics	-0.40	0.25	0.114	0.00
	Interaction	0.52*	0.26	0.044	0.00

Note. Estimates represent unstandardized regression weights in pre-intervention standard deviation units. Effect size is Cohen's f^2 and can be interpreted as small = 0.02, medium = 0.15, large = 0.35.

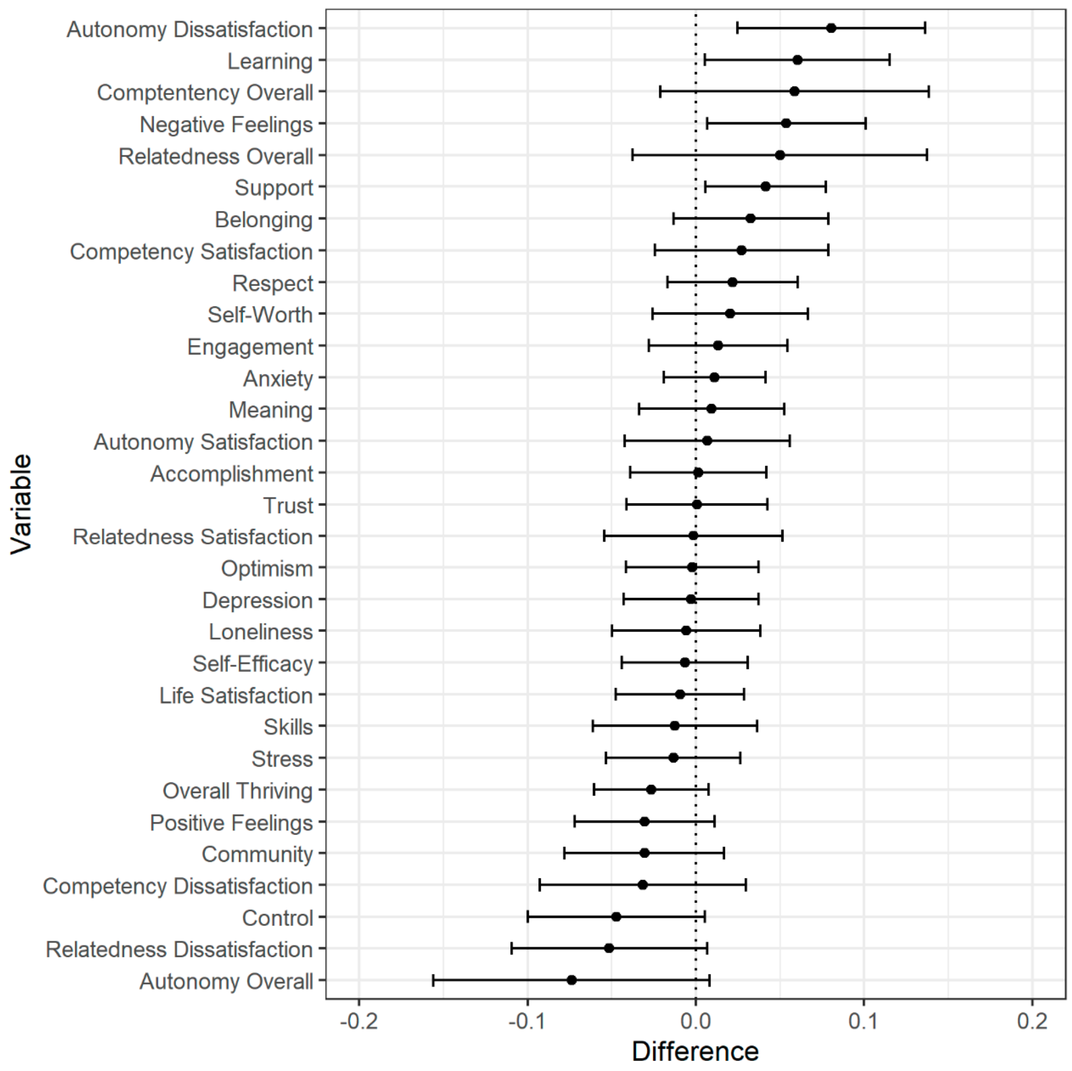

Supplementary Figure S1. Changes in well-being indices before and after the intervention.

Note. Estimates are differences (Post – Pre) in scale units with 95% confidence intervals. Positive values indicate increases in the outcome; negative values indicate decreases in the outcome.

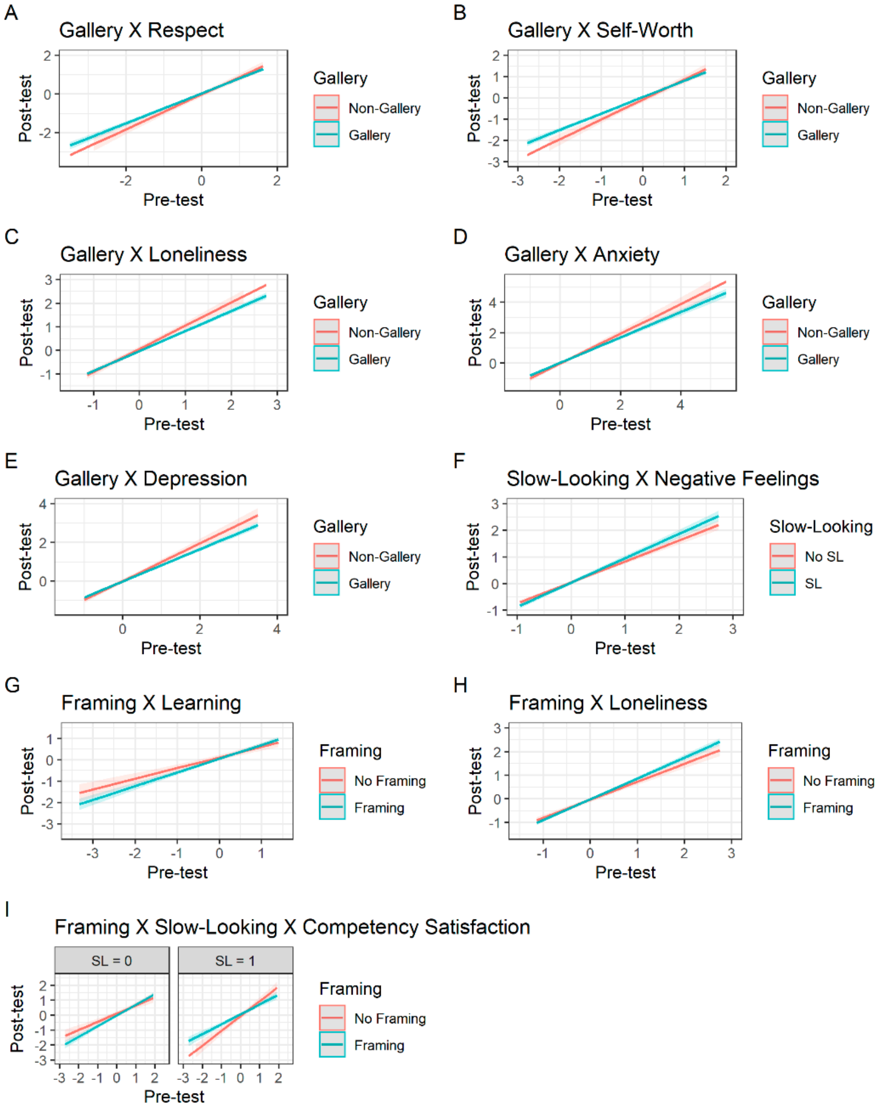

Supplementary Figure S2. Interactions between experimental condition and pre-test flourishing predicting post-test flourishing
